# Preparation of Novel Homodimers Derived from Cytotoxic Isoquinolinequinones. A Twin Drug Approach

**DOI:** 10.3390/molecules23020439

**Published:** 2018-02-16

**Authors:** Juana Andrea Ibacache, Judith Faundes, Margarita Montoya, Sophia Mejías, Jaime A. Valderrama

**Affiliations:** 1 Facultad de Química y Biología, Universidad de Santiago de Chile, Alameda 3363, Casilla 40, Santiago 9170022, Chile; judith.faundes@usach.cl (J.F.); margarita.montoya@usach.cl (M.M.); sophia.mejias@usach.cl (S.M.); 2 Facultad de Ciencias de la Salud, Universidad Arturo Prat, Casilla 121, Iquique 1100000, Chile

**Keywords:** anilinoisoquinolinequinones, twin drugs, homodimers, amination reaction, cytotoxic activity

## Abstract

The synthesis of five novel homodimers is reported based on the anilinoisoquinolinequinone scaffold. In these twin-drug derivatives, two units of the anilinoquinone pharmacophores are linked through a methylene spacer. The formation of dimers was achieved by reaction of isoquinolinequinones with 4, 4′-diaminodiphenylmethane via a sequence of two oxidative amination reactions. A preliminary in vitro screening of the homodimers reveals moderate to high cytotoxic activities against MDA-MB-21 breast adenocarcinoma and B16-F10 murine metastatic melanoma cell lines. The asymmetrical homodimer **15** stands out due to its cytotoxic potencies at submicromolar concentrations and high selectivity index (mean IC_50_ = 0.37 μM; SI = 6.97) compared to those of etoposide (mean IC_50_ = 3.67; SI = 0.32) and taxol (mean IC_50_ = 0.35; SI = 0.91) employed as reference anticancer drugs.

## 1. Introduction

**Figure 1:**
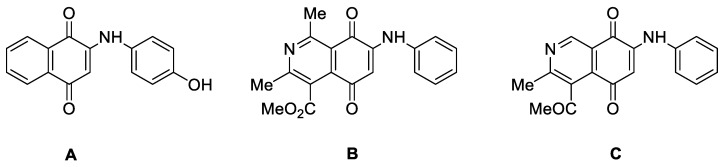
Examples of cytotoxic anilino-1,4-naphthoquinone and hetero-analogues.

The quinone nucleus is the common feature of many drugs used clinically in the therapy of solid cancers, such as daunorubicin, mitomycin, mitoxantrone, and saintopin. The most remarkable characteristics of quinonoid compounds are their ability to acts as DNA intercalators, reductive alkylators of biomolecules, and/or generators of reactive oxygen species (ROS), which can damage tumor cells [1,2,3,4,5,6]. Since many of the currently available anticancer drugs are incapable of differentiating between normal and neoplastic cells, there is a pressing need for new anticancer agents with high potency and less toxicity to noncancerous cells. Among the broad variety of synthetic quinonoid compounds, a group of donor–acceptor members derived from 2-anilino-1,4-naphoquinone and 7-anilinoisoquinoline-5,8-quinone analogues such as compounds A, B and C (Figure 1), have a wide range of remarkable in vitro cytotoxic activity against a variety of cancer cell lines [7,8,9,10,11].

We have recently reported preliminary synthetic efforts aimed at the construction of homodimers of 7-anilinoisoquinolinequinones such as B and C, to produce twin drugs of these cytotoxic pharmacophores [12]. The linkage of two identical pharmacophoric entities, generating an “identical twin drug” or homodimer derivative, is a classical strategy used in medicinal chemistry to produce more potent and/or selective drugs compared to the single entities [13,14,15,16]. Our efforts to prepare homodimers from isoquinolinequinones and symmetric aryldiamines such as *p*-phenylendiamine, benzidine, and dapsone were unsuccessful, since the diamines act as mononucleophiles to produce the corresponding arylaminoisoquinolinequinones [12]. The lack of reactivity of these amination products to undergo a further amination reaction with the isoquinolinequinones to give the corresponding homodimers is probably due to significant donor–acceptor interactions between the quinone and the arylamino components [17], thus decreasing the nucleophilic character of the NH_2_ groups. Here, we report successful results on the access to five homodimers based on two identical anilinoisoquinolinequinone fragments linked via a methylene spacer, from isoquinolinequinones and 4, 4′-diaminodiphenylmethane. The preparation of monoamination compounds as valuable precursors of heterodimers is also described. A preliminary in vitro evaluation of the new homodimers and their corresponding arylaminoisoquinolinequinones intermediates is reported.

## 2. Results and Discussion

### 2.1. Chemistry

**Figure 2:**
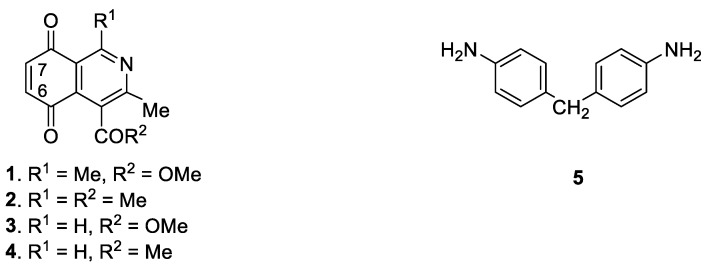
Starting compounds for the target homodimers.

The isoquinolinequinones **1**–**4** and the commercially available symmetrical diamine **5**, were selected as starting materials to prepare the target identical twin-drug homodimer types containing the anilinoisoquinolinequinone pharmacophores B and C (Figure 2). Quinones **1**–**4** were synthesized by a previously reported method [9,10].

**Scheme 1:**
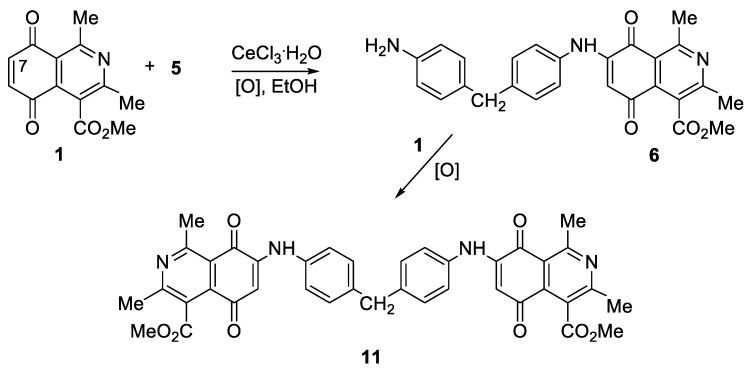
Products from the reaction of quinone **1** with diamine **5**.

We first examined the reaction of quinone **1** with diamine **5** in a 2:1 mole ratio in the presence of catalytic amounts of CeCl_3_ × 7H_2_O in ethanol at room temperature. Work-up of the reaction mixture followed by column chromatography yielded product **6** and the symmetric homodimer **11** in 8 and 41% yields, respectively (Scheme 1).

The structures of compounds **6** and **11**, in which the quinoid moiety is bonded to the diamine through the 7-position, were fully characterized by infrared spectroscopy (IR), ^1^H- and ^13^C-nuclear magnetic resonance (NMR), bidimensional nuclear magnetic resonance (2D-NMR), and high resolution mass spectroscopy (HRMS).

It is evident that the reaction of quinone **1** with diamine **5** under aerobic conditions proceeds to give product **6**, which, by a further oxidative amination with electrophile **1**, yields homodimer **11**. The results show that the amino group of compound **6** is as nucleophilic as those of diamine **5** to react with electrophile **1**, to give **11**. It can therefore be concluded that there are no significant electronic interactions between the amino group and the donor–acceptor anilinoisoquinolinquinone fragment of compound **6**.

Based on this preliminary assay (Scheme 1), we focused on the selective access to the target symmetric homodimer **11** by performing the reaction of **1** with amine **5** in a 4:1 mole ratio under the above-mentioned conditions. Surprisingly, the reaction produced the expected dimer **11** in nearly quantitative yield (98%).

**Scheme 2:**
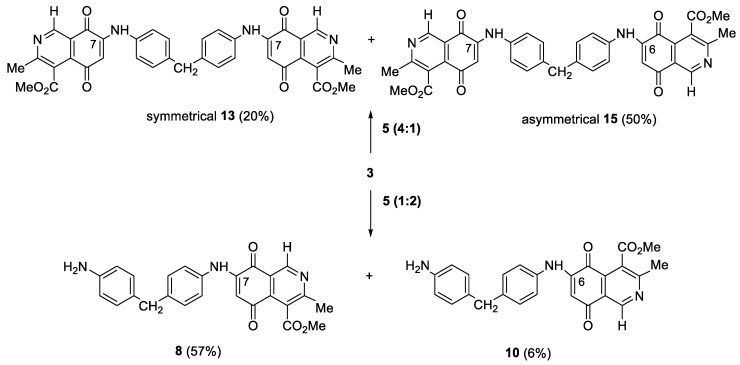
Formation of compounds **8**/**10** and homodimers **13**/**15** from **3** and **5**.

Taking into account the behavior of **6** to react with isoquinolinequinone **1** and its potential application in the synthesis of new heterodimers by reaction with different cytotoxic carbo- and heterocyclic quinones, we investigated the experimental conditions to allow selective access to compound **6**. After several trials, we found that **6** is formed in 74% yield by reaction of **1** with amine **5** in a 1:2 mole ratio under the standard conditions (Table 1). Based on the optimal experimental conditions to prepare compounds **6** and **11**, the synthesis of monoamination and homodimer products from isoquinolinequinones **2**–**4** and diamine **5** was attempted. The reactions of quinones **2**, **3**, and **4** with **5** in a 2:1 mole ratio produced the expected monoamination products **7**, **8**, and **9** in 55, 57 and 32% yield, respectively. From the reaction of quinone **3** with **5**, the monoamination compound **10** was isolated along with **8**, albeit in poor yield (6%) (Scheme 2; Table 2). The reaction of quinones **2**, **3** and **4** with **5** in a 4:1 mole ratio produced the expected homodimers **12**, **13**, and **14** in 95, 20, and 58% yield, respectively. From the reaction of quinone **3** with **5**, the asymmetrical homodimer **15** was isolated in 50% yield, along the symmetrical homodimer **13**, (Table 2). The findings on the reactions of quinone **3** with diamine **5** suggest that competitive nucleophilic attacks of compounds **8**/**10** to the 6- and 7-positions of quinone **3** are involved.

**Figure 3:**
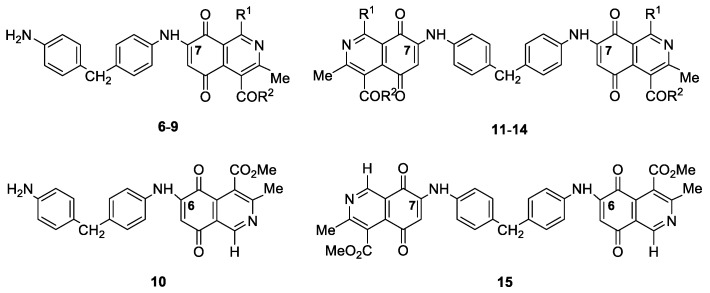
Structure of the monoamination and homodimer compounds **6**–**10** and **11**–**15**.

The results of the study on the preparation of the monoamination and homodimer compounds are shown in Figure 3 andTable 1.

It should be noted that the oxidative amination reaction of quinone **1** with diamine **5** produced homodimer **11** as the unique regioisomer; however, two isomeric homodimers, **13** and **15**, were generated in the reaction of quinone **3** with diamine **5**. The differences in the regiochemistry of the oxidative amination reaction of quinones **1** and **3** with amine **5**, are in agreement with previous studies on the oxidative amination of isoquinolinequinones with amines. For instance, it was determined that quinone **1** reacts with alkyl- and arylamines in a regiospecific manner to furnish the respective 7-substituted regioisomers [9,11]. In the case of quinone **3**, the amination reactions take place with regioselective preferences to give the 7-substituted regioisomers, as the main products, along with the 6-substituted regioisomers [18]. The regiochemical control of the substitution reactions of quinones **1** and **3** can be explained assuming stereoelectronic interactions between the substituents at C-1 (CH_3_ and H) and the carbonyl group at C-8. These factors probably affect the electrophilicity of the C-7 and the preference of the nitrogen nucleophiles to attack this electrophilic centre.

**Figure 4:**
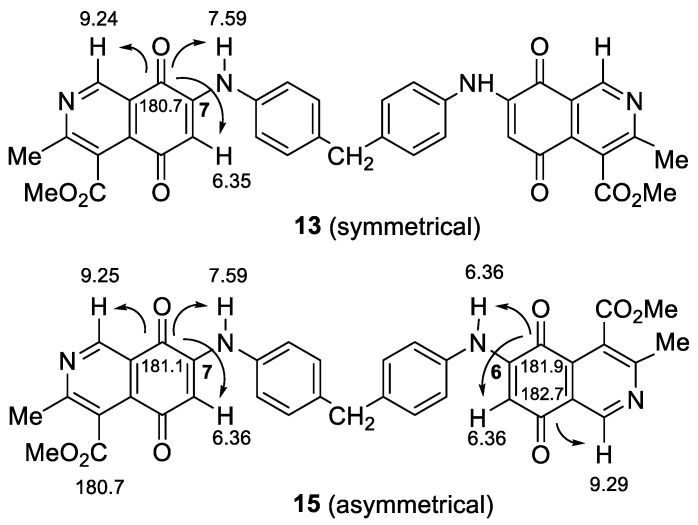
HMBC correlations of homodimers **13** and **15**.

The structures of the arylaminoquinones and homodimers were determined by IR, ^1^H-, ^13^C- NMR, 2D-NMR and HRMS. The symmetrical homodimers **11**–**14** showed magnetically equivalent spectroscopic signal patterns, indicating their C2-symmetric molecular feature in solution. Heteronuclear multiple bond correlation (HMBC) experiments were used to establish the structure of symmetrical and asymmetrical homodimers **13** and **15** (Figure 4).

### 2.2. Biological Results

The prepared homodimers **11**–**15** were evaluated for their in vitro cytotoxic activity using a conventional fluorescence assay (Cyquant direct cell proliferation assays) [19], against primary mouse embryo fibroblast cell line MEF and two cancer cells lines: MDA-MB-21 human breast adenocarcinoma and B16-F10 murine metastatic melanoma cells, in 72 h drug exposure assays. Monoamination products **6**–**10** were included in these preliminary assays. Table 2 shows the IC_50_ values, and selectivity indexes of the new compounds. Etoposide and taxol, used clinically as anticancer agents, were taken as positive controls.

According to the data in Table 2, compounds **14** and **15** appeared as the most potent members of the synthesized homodimers. Compound **15** stands out due to its cytotoxic activity at submicromolar concentrations and high selectivity index (mean IC_50_ = 0.37 μM; SI = 6.97) compared to those of etoposide (mean IC_50_ = 3.67; SI = 0.32) and taxol (mean IC_50_ = 0.35; SI = 0.91) used as reference anticancer drugs.

In view of the high incidence of undesirable side effects induced by the majority of current anticancer drugs and considering the selective indexes of homodimers **14** and **15**, they appear as promising and interesting leads, having potential anticancer activity.

## 3. Materials and Methods

### 3.1. General

All solvents and reagents were purchased from different companies such as Aldrich (St. Louis, MO, USA) and Merck (Darmstadt, Germany) and were used as supplied. Melting points were determined on a Stuart Scientific SMP3 (Bibby Sterilin Ltd., Staffordshire, UK) apparatus and are uncorrected. The IR spectra were recorded on an FT IR Bruker spectrophotometer; (model Vector 22 Bruker, Rheinstetten, Germany), using KBr disks, and the wave numbers are given in cm^−1^. ^1^H- and ^13^C-NMR spectra were recorded on a Bruker Avance-400 instrument (Bruker, Ettlingen, Germany) in CDCl_3_ at 400 and 100 MHz, respectively. Chemical shifts are expressed in ppm downfield relative to tetramethylsilane and the coupling constants (*J*) are reported in hertz. Data for ^1^H-NMR spectra are reported as follows: s = singlet, br s = broad singlet, d = doublet, m = multiplet, and the coupling constants (*J*) in Hz. Bidimensional NMR techniques were used for signal assignments. HRMS-ESI were carried out on a Thermo Scientific Exactive Plus Orbitrap spectrometer (Bremen, Germany) with a constant nebulizer temperature of 250 °C. The experiments were performed in positive ion mode, with a scan range of *m*/*z* 100–300. All fragment ions were assigned by accurate mass measurements at high resolution (resolving power: 140,000 FWHM). The samples were infused directly into the electrospray ionization source (ESI) using a syringe pump at flow rates of 5 μL min^−1^. Silica gel Merck 60 (70–230 mesh, from Merck, Darmstadt, Germany) was used for preparative column chromatography, and TLC aluminum foil 60F254 for analytical thin layer chromatography (TLC). Isoquinolinequinones **1**–**4** were prepared by previously reported procedures [9,11].

### 3.2. Chemistry

#### Preparation of Compounds **6**–**10** and Homodimers **11**–**15**, General Procedure

Suspensions of quinones **1**–**4** and 4,4′-diaminodiphenylmethane **5**, CeCl_3_ × 7H_2_O (5% mmol with respect to the limiting reagent 1 or 4) and ethanol (20 mL) were left with stirring at RT after completion of the reaction as indicated by TLC. The solvents were removed under reduced pressure and the residues were column cromatographed over silica gel (95:5 CH_2_Cl_2_/EtOAc) to yield the corresponding pure compounds **6**–**10** or the homodimers **11**–**1**5.

*Methyl-7-(4-(4-aminobenzyl)phenyl)amino)-1,3-dimethyl-5,8-dioxo-5,8-dihydroisoquinoline-4-carboxylate* (**6**). Prepared in 74% yield (4 h, 66.8 mg, 0.15 mmol,) from quinone **1** (50 mg, 0.20 mmol), and **5** (80.9 mg, 0.41 mmol); red solid, m.p.: 149–150 °C; IR (KBr): ν_max_: 3423 (N-H), 3305 and 3251 (N-H), 1734 (C=O ester), 1617 and 1600 (C=O quinone). ^1^H-NMR (CDCl_3_) δ 2.61 (s, 3H, 3-Me), 2.99 (s, 3H, 1-Me), 3.60 (s, 2H, NH_2_), 3.88 (s, 2H, CH_2_), 4.00 (s, 3H, CO_2_Me), 6.30 (s, 1H, 6-H), 6,62 (dd, *J* = 8.3 Hz, 12.8 Hz, 2H), 6.96 (t, *J =* 6.8 Hz, 2H), 7.14 (d, *J =* 8.3 Hz, 2H), 7.22 (d, *J* = 8.3 Hz, 2H), 7.68 (s, 1H, N-H). ^13^C-NMR (CDCl_3_) δ 182.1, 181.7, 169.6, 161.6, 161.3, 146.0, 145.1, 142.7, 140.8, 138.3, 130.9, 130.5, 125.5, 123.4, 120.3, 115.8, 115.7, 102.5, 53.4, 40.9, 26.5, 23.3. HRMS [M+H]^+^: calcd for C_26_H_23_N_3_O_4_: 442.1762; found: 442.1761.

*4-Acetyl-7-(4-(4-aminobenzyl)phenyl)amino)-1,3-dimethylisoquinoline-5,8-dione* (**7**). Prepared in 55% yield (4 h, 51.4 mg, 0.12 mmol) from quinone **2** (50 mg, 0.22 mmol), and **5** (86.3 mg, 0.44 mmol); red solid, m.p.: 100–101 °C; IR (KBr): ν_max_: 3433 (N-H), 3355 and 3245 (NH_2_), 1516 (C=O acetyl), 1619 and 1598 (C=O quinone). ^1^H-NMR (CDCl_3_) δ 2.52 (s, 3H, COMe), 2.56 (s, 3H, 3-Me), 2.98 (s, 3H, 1-Me), 3.88 (s, 2H, CH_2_), 6.28 (s, 1H, 6-H), 6.65 (d, *J* = 8.1 Hz, 2H), 6.97 (d, *J* = 8.1, 2H), 7.15 (d, *J* = 8.2 Hz, 2H), 7.23 (d, *J* = 8.2 Hz, 2H), 7.70 (s, 1H, N-H). ^13^C-NMR (CDCl_3_) δ 204.0, 182.6, 182.1, 160.8, 160.2, 146.3, 145.1, 140.9, 138.2, 135.0, 133.9, 130.8, 130.5, 130.1, 123.5, 120.4, 115.8, 102.2, 40.9, 31.4, 26.3, 23.3.HRMS [M+H]^+^: calcd for C_26_H_23_N_3_O_3_: 426.1812; found: 426.1798.

*Methyl-7-(4-(4-aminobenzyl)phenyl)amino)-3-methyl-5,8-dioxo-5,8-dihydroisoquinoline-4-carboxylate* (**8**). Prepared in 57% yield (2 h, 104.6 mg, 0.24 mmol) from quinone **3** (100 mg, 0.43 mmol), and **5** (170 mg, 0.63 mmol); red solid, m.p.: 83–84 °C; IR (KBr): ν_max_: 3442 (N-H), 3302 and 3348 (N-H), 1731 (C=O ester), 1571 and 1514 (C=O quinone). ^1^H-NMR (CDCl_3_) δ 2.67 (s, 3H, 3-Me), 3.60 (s, 2H, NH_2_), 3.88 (s, 2H, CH_2_), 4.02 (s, 3H, CO_2_Me), 6.34 (s, 1H, 6-H), 6.64 (d, *J* = 8.2 Hz, 2H), 6.96 (d, *J* = 8.1 Hz), 7.15 (d, *J* = 8.3 Hz, 2H), 7.23 (d, *J* = 8.3 Hz, 2H), 7.57 (s, 1H, N-H), 9.24 (s, 1H, 1-H). ^13^C-NMR (CDCl_3_) δ 181.6, 181.2, 168.9, 163.4, 148.6, 145.3, 145.2, 141.0, 136.2, 134.9, 130.8, 130.5, 130.1, 126.4, 123.4, 122.2, 115.8, 103.8, 53.4, 40.9, 23.3. HRMS [M+H]^+^: calcd for C_25_H_21_N_3_O_4_: 428.1605; found: 428.1596.

*4-Acetyl-7-(4-(4-aminobenzyl)phenyl)amino)-3-methylisoquinoline-5,8-dione* (**9**). Prepared in 32% yield (6 h, 25 mg, 0.06 mmol) from quinone **4** (40.6 mg, 0.19 mmol), and **5** (74.4 mg, 0.38 mmol); red solid, m.p.: 167–168 °C; IR (KBr): ν_max_: 3468 (N-H), 3371 and 3289 (N-H), 1678 (C=O acetyl), 1616 and 1598 (C=O quinone). ^1^H-NMR (CDCl_3_) δ 2.56 (s, 3H, COMe), 2.61 (s, 3H, 3-Me), 3.67 (s, 2H, NH_2_), 3.88 (s, 2H, CH_2_), 6.33 (s, 1H, 6-H), 6.64 (d, *J* = 8.1 Hz, 2H), 6.97 (d, *J* = 8.1 Hz, 2H), 7.16 (d, *J* = 8.2 Hz, 2H), 7.24 (d, *J* = 8.2 Hz, 2H), 7.61 (s, 1H, N-H), 9.23 (s, 1H, 1-H). ^13^C-NMR (CDCl_3_) δ 204.1, 182.5, 181.2, 161.9, 148.2, 145.5, 145.2, 141.1, 136.1, 134.8, 134.7, 130.8, 130.5, 130.1, 123.5, 122.3, 115.8, 103.5, 40.9, 31.5, 23.3. HRMS [M+H]^+^: calcd for C_25_H_21_N_3_O_3_: 412.1656; found: 412.1649.

*4-Acetyl-6-((4-(4-aminobenzyl)phenyl)amino)-3-methylisoquinoline-5,8-dione* (**10**). Prepared in 6% yield (6 h, 4.4 mg, 0.01 mmol) from quinone **3** (40.6 mg, 0.19 mmol), and **5** (74.4 mg, 0.38 mmol); red solid, m.p.: 138–139 °C; IR (KBr): ν_max_: 3414 (N-H), 3352 and 3242 (N-H), 1515 (C=O acetyl), 1597 and 1568 (C=O quinone). ^1^H-NMR (CDCl_3_) δ 2.61 (s, 3H, COMe), 2.62 (s, 3H, 3-Me), 3.65 (s, 2H, NH_2_), 3.88 (s, 2H, CH_2_), 6.34 (s, 1H, 6-H), 6.64 (d, *J* = 8.2 Hz, 2H), 6.97 (s, *J* = 8.1 Hz, 2H), 7.15 (d, *J* = 8.9 Hz, 2H), 7.23 (d, *J* = 8.3 Hz, 2H), 7.30 (s, 1H, N-H), 9.27 (s, 1H, 1-H). ^13^C-NMR (CDCl_3_) δ 204.1, 182.9, 159.1, 148.5, 145.2, 140.9, 134.9, 134.0, 132.3, 130.5, 130.2, 123.5, 123.2, 116.1, 103.8, 40.9, 31.4, 30.1, 23.3, 22.9. HRMS [M+H]^+^: calcd for C_25_H_21_N_3_O_3_: 412.1656; found: 412.1661.

*Dimethyl-7,7′-(4,4′-methylenebis(4,1-phenylene)bis(azanediyl)bis(1,3-dimethyl-5,8-dioxo-5,8-dihydroisoquinoline-4-carboxylate)* (**11**). Prepared in 98% yield (40 h, 40.8 mg, 0.06 mmol) from quinone **1** (60 mg, 0.25 mmol), and **5** (12 mg, 0.06 mmol); red solid, m.p.: 199–200 °C; IR (KBr): ν_max_: 3446 (N-H), 1736 (C=O ester), 1618 and 1602 (C=O quinone). ^1^H-NMR (CDCl_3_) δ 2.64 (s, 6H, 3-Me), 3.02 (s, 6H, 1-Me), 4.03 (s, 8H, CH_2_ and CO_2_Me), 6.34 (s, 1H, 6-H), 7.26 (m, 8H, arom.), 7.73 (s, 2H, N-H). ^13^C-NMR (CDCl_3_) δ 181.6, 181.4, 169.1, 161.3, 160.9, 145.5, 138.8, 137.8, 135.2, 130.2, 125.1, 123.2, 119.9, 102.3, 53.0, 40.8, 26.1, 22.93. HRMS [M+H]^+^: calcd for C_39_H_32_N_4_O_8_: 685.2293; found: 685.2208.

*7,7′-(4,4′-Methylenebis(4,1-phenylene)bis(azanediyl))bis(4-acetyl-1,3-dimethylisoquinoline-5,8-dione)* (**12**). Prepared in 95% yield (32 h, 67.9 mg, 0.10 mmol) from quinone **2** (100 mg, 0.44 mmol), and **5** (21.6 mg, 0.11 mmol); red solid, m.p.: 272–273 °C; IR (KBr): ν_max_: 3446 (N-H), 1519 (C=O acetyl), 1593 and 1564 (C=O quinone). ^1^H-NMR (CDCl_3_) δ 2.52 (s, 6H, COMe), 3.02 (s, 6H, 3-Me), 2.99 (s, 6H, 1-Me), 4.01 (s, 2H, CH_2_), 6.30 (s, 2H, 6-H), 7.21 (m, 8H, arom.), 7.72 (s, 2H, N-H). ^13^C-NMR (CDCl_3_): δ 204.15, 182.72, 182.00, 160.88, 160.25, 146.15, 139.32, 138.14, 135.49, 133.93, 130.64, 123.73, 120.35, 102.33, 41.24, 31.49, 26.34, 23.30. HRMS [M+H]^+^: calcd for C_39_H_32_N_4_O_6_: 653.2395; found: 653.2365.

*Dimethyl-7,7′-(4,4′-methylenebis(4,1-phenylene)bis(azanediyl))bis(3-methyl-5,8-dioxo-5,8-dihydroisoquinoline-4-carboxylate)* (**13**). Prepared in 20% yield (32 h, 8.4 mg, 0.01 mmol) from quinone **3** (60 mg, 0.26 mmol), and **5** (13 mg, 0.07 mmol); red solid, IR (KBr): ν_max_: 3446 (N-H), 1724 (C=O ester), 1600 and 1573 (C=O quinone). ^1^H-NMR (CDCl_3_) δ 2.66 (s, 6H, 3-Me), 4.01 (s, 8H, CO_2_Me and CH_2_), 6.35 (s, 2H, 6-H), 7.22 (m, 8H, arom.), 7.59 (s, 2H, N-H), 9.24 (s, 2H, 1-H). ^13^C-NMR (CDCl_3_) δ 181.3, 180.7, 168.6, 163.1, 148.2, 144.7, 138.9, 135.7, 134.9, 130.3, 126.0, 123.2, 121.8, 103.5, 53.1, 40.8, 23.0. HRMS [M+H]^+^: calcd for C_37_H_28_N_4_O_8_: 657.1980; found: 657.1965.

*7,7′-(4,4′-Methylenebis(4,1-phenylene)bis(azanediyl))bis(4-acetyl-3-methylisoquinoline-5,8-dione)* (**14**). Prepared in 58% yield (32 h, 33.8 mg, 0.05 mmol) from quinone **4** (80 mg, 0.32 mmol), and **5** (18 mg, 0.09 mmol); red solid, m.p.: 217–218 °C; IR (KBr): ν_max_: 3446 (N-H), 1521 (C=O acetyl), 1598 and 1568 (C=O quinone). ^1^H-NMR (CDCl_3_) δ 2.56 (s, 6H, COMe), 2.61 (s, 6H, 3-Me), 4.02 (s, 2H, CH_2_), 6.35 (s, 2H, 6-H), 7.24 (m, 8H, arom.), 7.63 (s, 2H, N-H), 9.23 (s, 2H, 1-H). ^13^C-NMR (CDCl_3_) δ 204.1, 182.6, 181.2, 162.1, 148.2, 145.4, 139.4, 136.0, 135.3, 134.8, 130.7, 123.7, 122.3, 103.6, 41.2, 31.5, 23.3. HRMS [M+H]^+^: calcd for C_37_H_28_N_4_O_6_: 625.2082; found: 625.2083.

*Methyl-7-(4-(4-(4-(methoxycarbonyl)-3-methyl-5,8-dioxo-5,8-dihydroisoquinolin-6-ylamino)benzyl)phenylamino)-3-methyl-5,8-dioxo-5,8-dihydroisoquinoline-4-carboxylate* (**15**). Prepared in 50% yield (26 h, 36.3 mg, 0.06 mmol) from quinone **4** (102 mg, 0.44 mmol), and **5** (21.9 mg, 0.11 mmol); purple solid, m.p.: 191–192 °C; IR (KBr): ν_max_: 3398 (N-H), 1736 (C=O ester), 1598 and 1572 (C=O quinone). ^1^H-NMR (CDCl_3_) δ 2.67 (s, 6H, 3-Me), 4.01 (s, 2H, CH_2_), 4.02 (s, 3H, CO_2_Me), 4.07 (s, 3H, CO_2_Me), 6.36 (s, 2H, 6-H), 7.21 (m, 8H, arom.), 7.36 (s, 1H, N-H), 7.59 (s, 1H, N-H), 9.25 (s, 1H, 1-H), 9.29 (s, 1H, NH). ^13^C-NMR (CDCl_3_) δ 182.7, 181.9, 181.7, 181.1, 168.9, 168.6, 163.5, 160.8, 149.0, 148.6, 145.2, 139.4, 139.2, 136.1, 135.5, 135.4, 132.6, 130.7, 126.4, 125.4, 123.7, 123.7, 122.9, 122.2, 104.0, 103.9, 53.6, 53.5, 41.2, 23.4, 23.0. HRMS [M+H]^+^: calcd for C_37_H_28_N_4_O_8_: 657.1980; found: 657.1991.

### 3.3. Cell Growth Inhibition Assay

The cell lines used in this work included MDA-MB-231 human breast adenocarcinoma cells, B16-F10 mouse melanoma cells and MEF primary mouse embryonic fibroblasts. Cells were grown in DMEM high glucose medium (Mediatech, Manassas, VA, USA) supplemented with 10% (MDA-MB-231, and B16-F10) or 15% (MEF) heat-inactivated fetal bovine serum (HyClone laboratories, South Logan, UT, USA), 100 IU/mL penicillin and 100 μg/mL streptomycin, kept at 37 °C in a 5% CO_2_ humidified atmosphere. For the experiments, a total of 5.000 cells/well were seeded on a flat-bottomed 96-well plate with 200 μL final volume. Six hours after seeding, the cells were incubated with the medium containing the compounds at concentrations ranging from 0 up to 100 μM dissolved in DMSO (0.1% final concentration) for 72 h. The concentrations used to calculate the IC_50_ values were 100.0, 30.0, 10.0, 3.0, 1.0, 0.3, 0.1, 0.01 and 0.0 μM. Untreated cells (medium containing 0.1% DMSO) were used as controls. At the end of the incubation, cell viability was measured using CyQuant® direct cell proliferation assay kits (Life Technologies, Carlsbad, CA, USA) in accordance with the manufacturer’s instructions. Briefly, 100 μL of culture medium containing the compounds under evaluation was removed from each well and replaced by 2X detection reagent. The cells were incubated for 1 h and fluorescence emission was measured at 535 nm with excitation at 480 nm in a microplate reader (Infinite 200 PRO, Tecan, Männedorf, Switzerland). At least four independent experiments were performed for each concentration. Each result was transformed to percentage of controls and the IC_50_ values were obtained graphically from the dose–response curves. The IC_50_ value was obtained by adjusting the dose–response curve to a sigmoidal curve (variable slope) generated using GraphPad Prisma 6.0 software (La Jolla, CA, USA).

## 4. Conclusions

In conclusion, we have prepared new homodimers and monoamination compounds derived from cytotoxic isoquinolinequinones and the symmetrical 4.4′-diaminodiphenylmethane. Selective access to these compounds was achieved through a one-step procedure, using appropriate reactant ratios. The high cytotoxic potencies of the usymmetrical homodimer **15** and the potential application of the monoamination compounds to prepare new heterodimers by combining different cytotoxic anilinoquinones, opens the possibility of constructing new twin drug scaffolds as more active and selective anticancer agents.

## Figures and Tables

**Table 1:** Yields of monoamination and homodimer compounds **6**–**10** and **11**–**15**.

Compound N°	R^1^	R^2^	Yield(%) *	N°	R^1^	R^2^	Yield(%) *
6	Me	OMe	74	11	Me	OMe	98
7	Me	Me	55	12	Me	Me	95
8	H	OMe	57	13	H	OMe	20
9	H	Me	32	14	H	Me	58
10	-	-	6	15	-	-	50

* Isolated by column chromatography.

**Table 2:** IC_50_ values, selectivity indexes of homodimers **11**–**15** and monoamination products **6**–**10**.

IC_50_ ± SEM (µM) ^a^					
Compound N	MEF ^b^	MDA-MB 231 ^c^	B16-F10 ^d^	Mean IC_50_	SI ^e^
Homodimer					
11	105.20 ± 13.86	66.90 ± 6.13	640 ± 12.66	353.45	0.30
12	71.56 ± 6.39	55.65 ± 5.95	48.61 ± 4.42	52.13	1.37
13	79.76 ± 9.27	24.81 ± 4.78	66.28 ± 5.61	45.55	1.75
14	32.75 ± 3.38	7.98 ± 1.43	5.83 ± 0.73	6.91	4.74
15	2.58 ± 0.33	0.29 ± 0.05	0.45 ± 0.08	0.37	6.97
**Monoamination product**					
6	8.87 ± 0.88	2.46 ± 0.39	6.16 ± 0.88	4.31	2.06
7	15.36 ± 1.97	19.54 ± 1.46	7.14 ± 0.95	13.34	1.15
8	5.54 ± 0.82	1.45 ± 0.31	3.31 ± 0.47	2.38	2.33
9	2.79 ± 0.44	1.59 ± 0.37	0.76 ± 0.21	1.18	2.36
10	5.14 ± 0.69	2.75 ± 0.42	2.17 ± 0.37	2.46	2.09
Etoposide	1.18 ± 0.40	5.34 ± 0.12	2.00 ± 0.44	3.67	0.32
Taxol	0.32 ± 0.05	0.32 ± 0.07	0.38 ± 0.06	0.35	0.91

^a^ Data represent average values of six independent determinations. ^b^ Normal mouse embryo fibroblast cell line. ^c^ Human breast adenocarcinoma cell line. ^d^ Murine metastatic melanoma cell line. ^e^ Mean selectivity index = IC_50_ values for fibroblast cells/ IC_50_ values tumor for cells.

## Supplementary Materials

The ^1^H-NMR, ^13^C-NMR, 2D-NMR spectra of compounds **6**–**15** are available as supporting data. Supplementary materials are available online.
