# Supplementary material for: Preparation of Novel Homodimers Derived from Cytotoxic Isoquinolinequinones. A Twin Drug Approach
Source: Molecules. 2018 Feb 16;23(2):439. doi: 10.3390/molecules23020439 (PMC6100386; doi:10.3390/molecules23020439)
Supplement: Supplementary file 1 [file molecules-23-00439-s001.pdf]

Supporting Information

Preparation of Novel Homodimers derived from Cytotoxic Isoquinolinequinones. A twin drug Approach

Juana Andrea Ibacache [1,*], Judith Faundes [1], Margarita Montoya[1], Sophia Mejías[1] and Jaime A. Valderrama [2,*]

[1] Facultad de Química y Biología, Universidad de Santiago de Chile, Alameda 3363, Casilla 40, Santiago 9170022, Chile; juana.ibacache.r@usach.cl
[2] Facultad de Ciencias de la Salud, Universidad Arturo Prat, Casilla 121, Iquique 1100000, Chile; jaimeadolfov@gmail.com
* Correspondence: juana.ibacache.r@usach.cl; Tel.: +56-02-718-1145

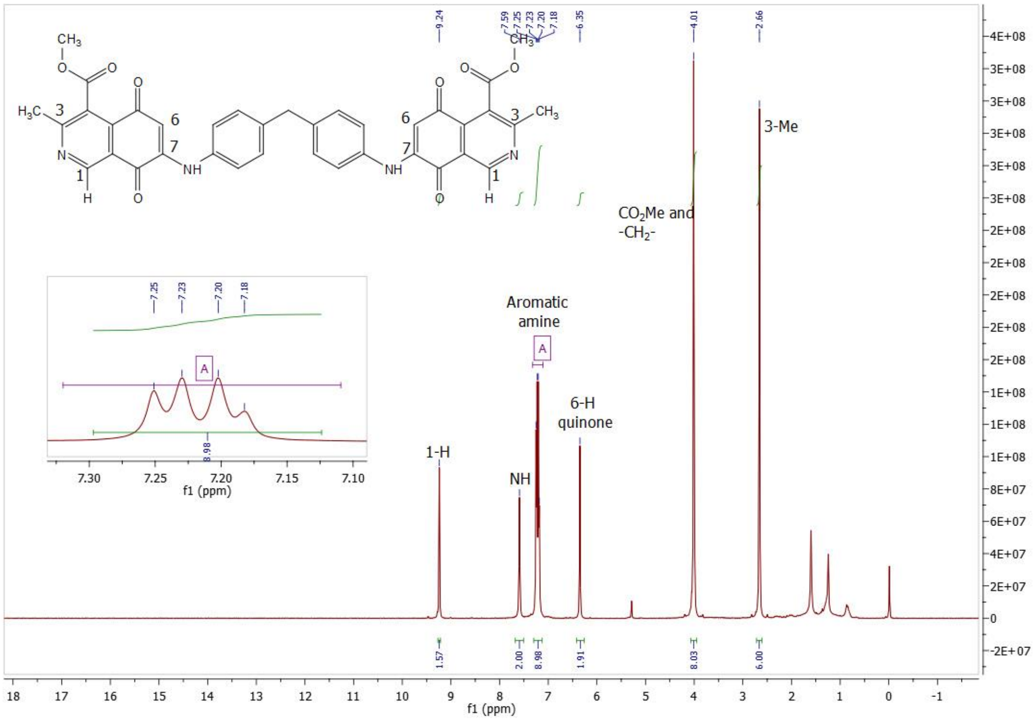

Figure S1. ^{1}H-NMR spectrum of compound **13**

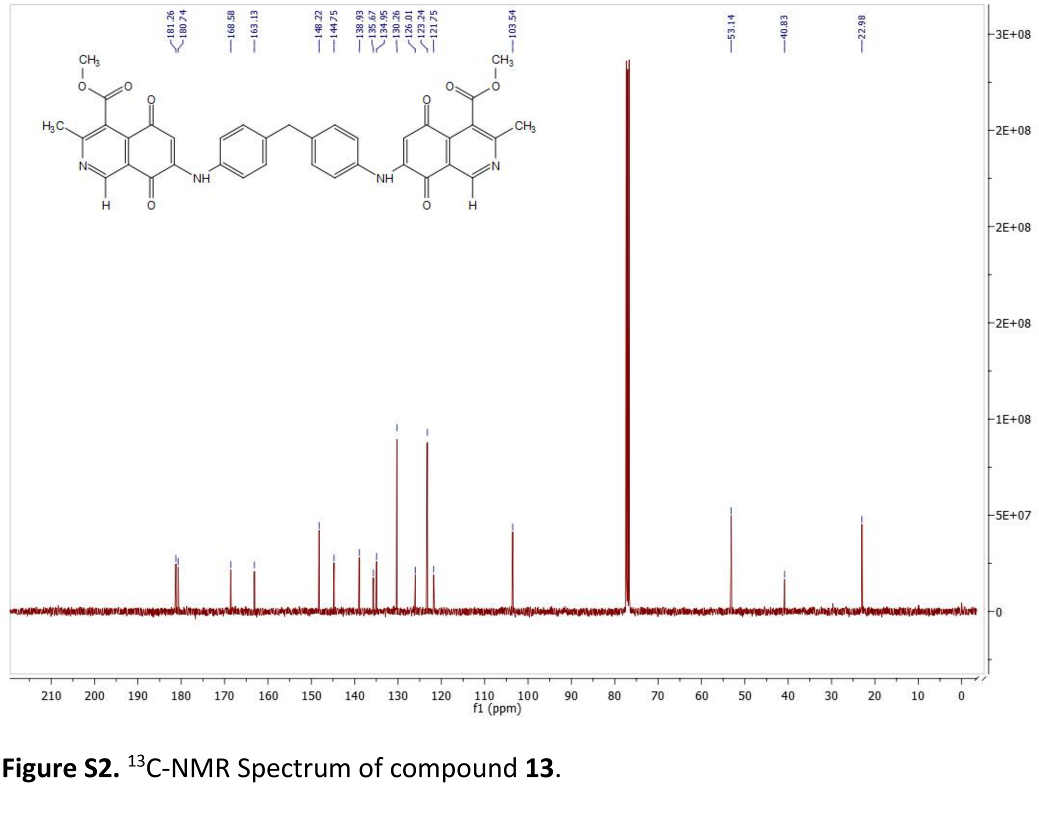

Figure S2. ^{13}C-NMR Spectrum of compound **13**.

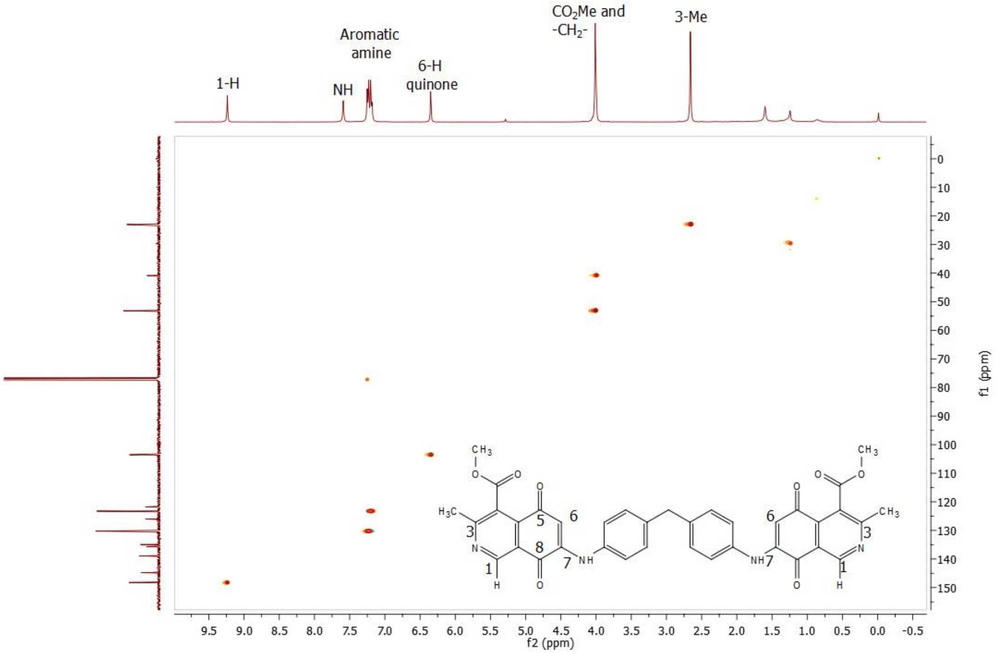

Figure S3. HSQC spectrum of compound **13**.

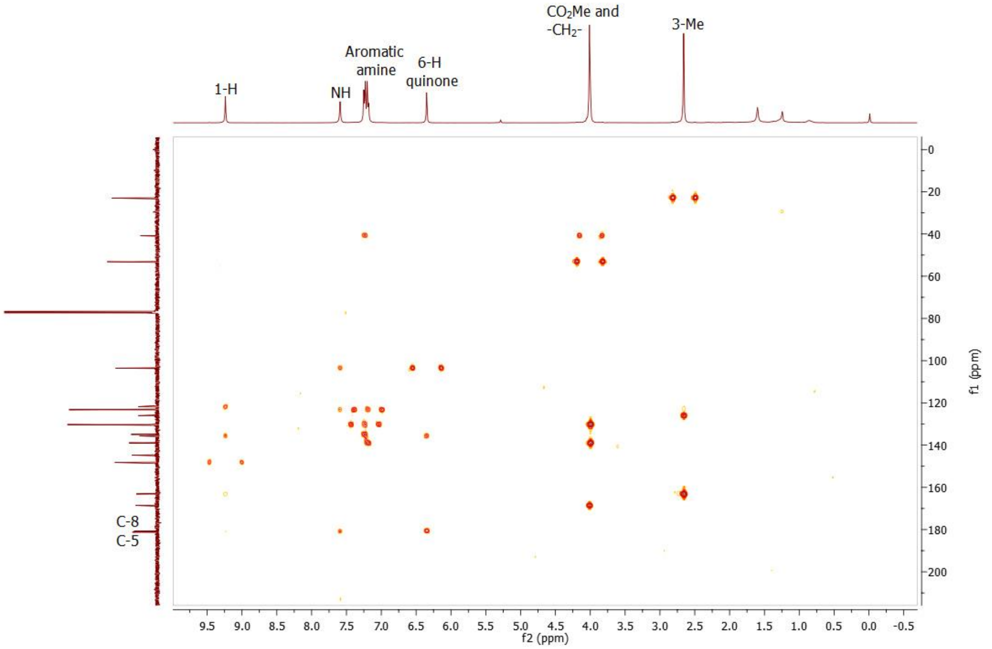

Figure S4. HMBC spectrum of compound **13**.

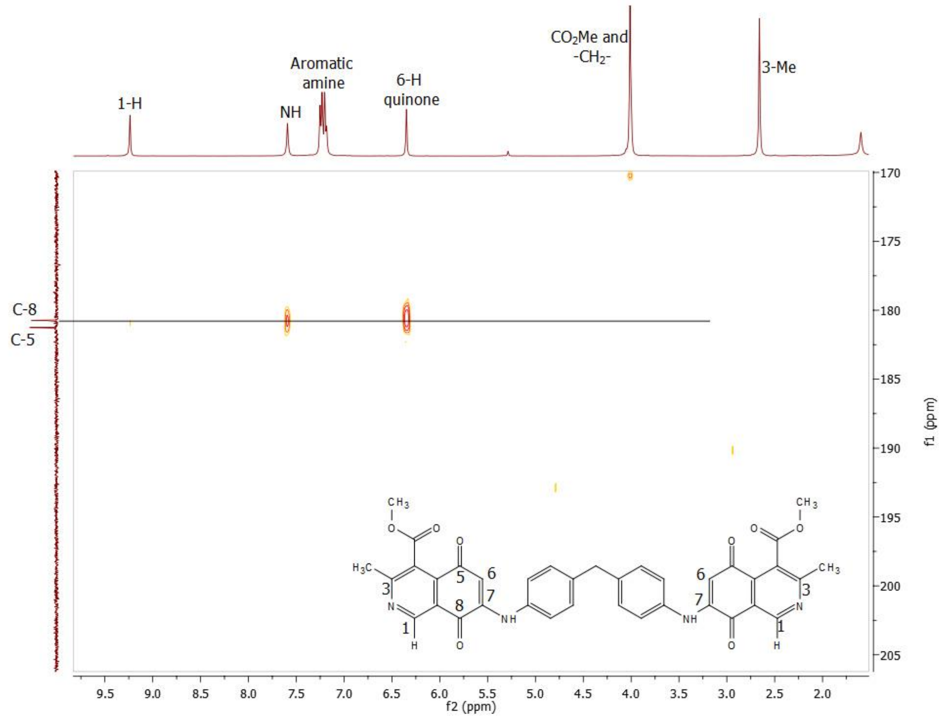

Figure S5. Expanded HSQC spectrum view of compound **13**.

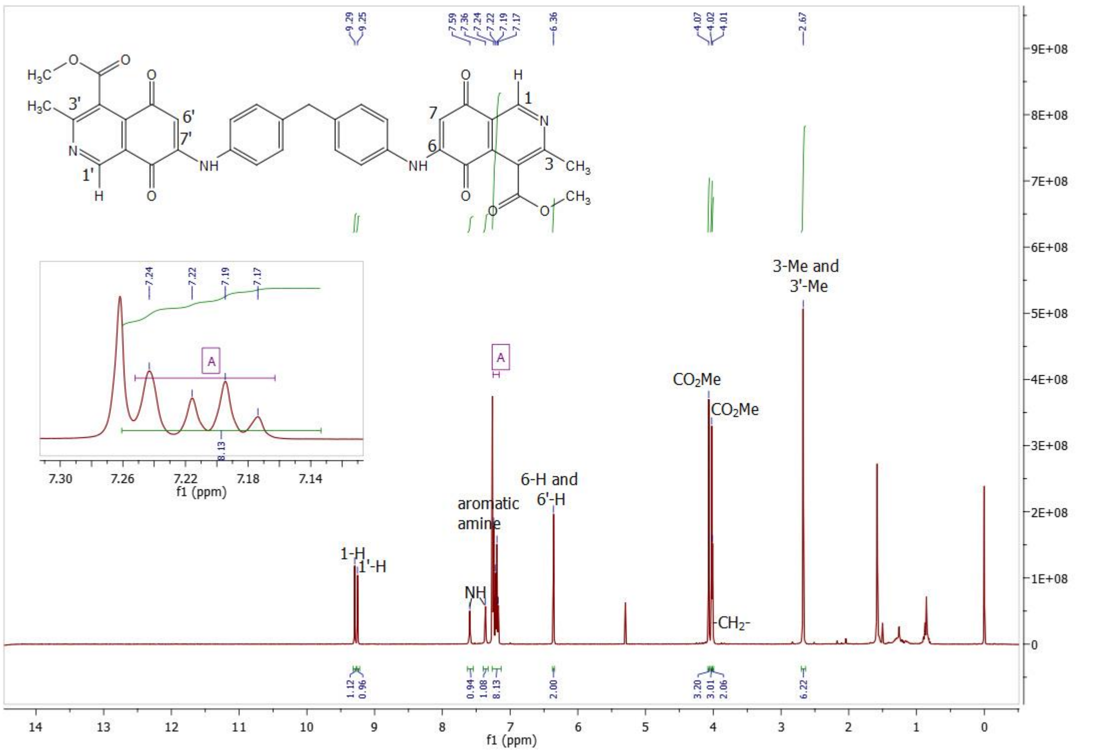

Figure S6. ^{1}H-NMR spectrum of compound **15**.

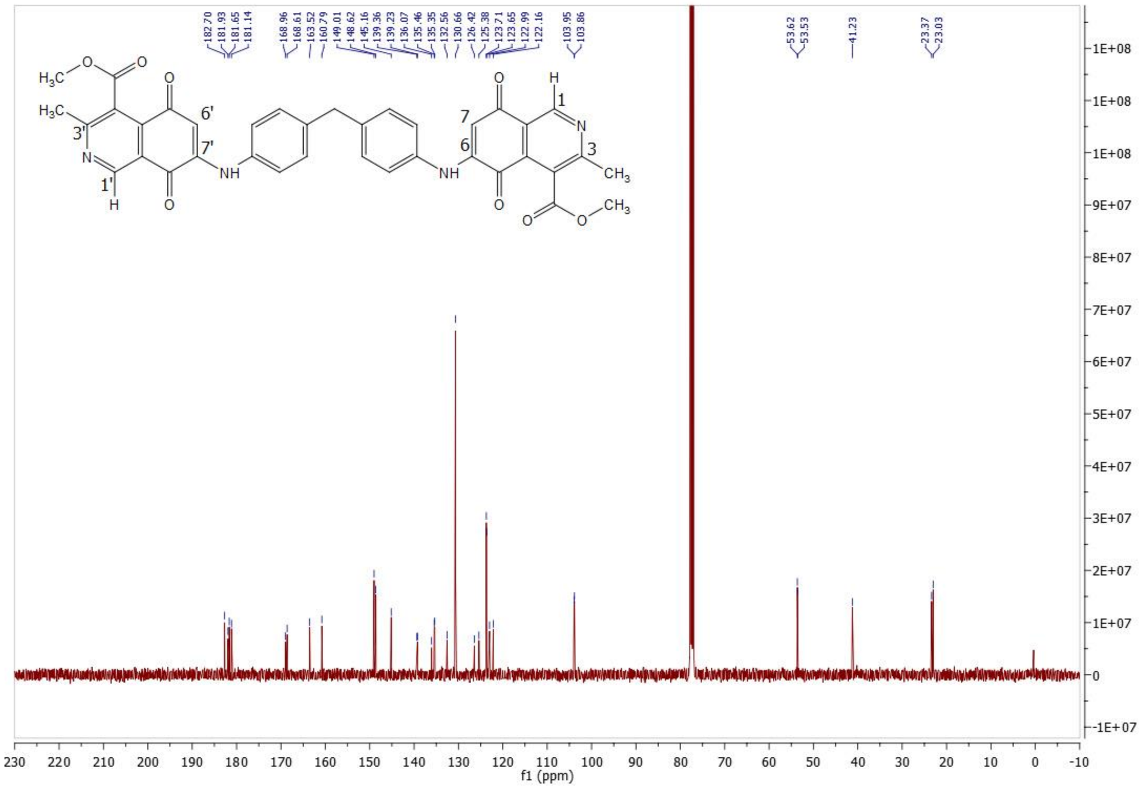

Figure S7. ^{13}C-NMR spectrum of compound **15**.

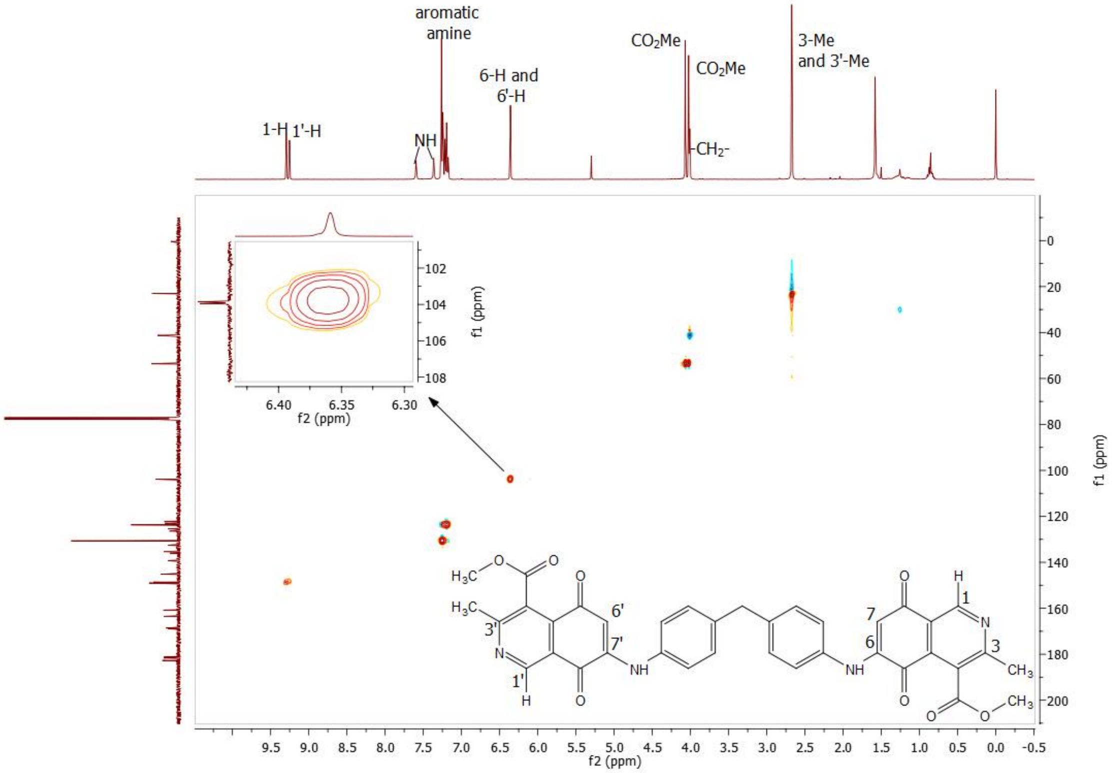

Figure S8. HSQC spectrum of compound **15**.

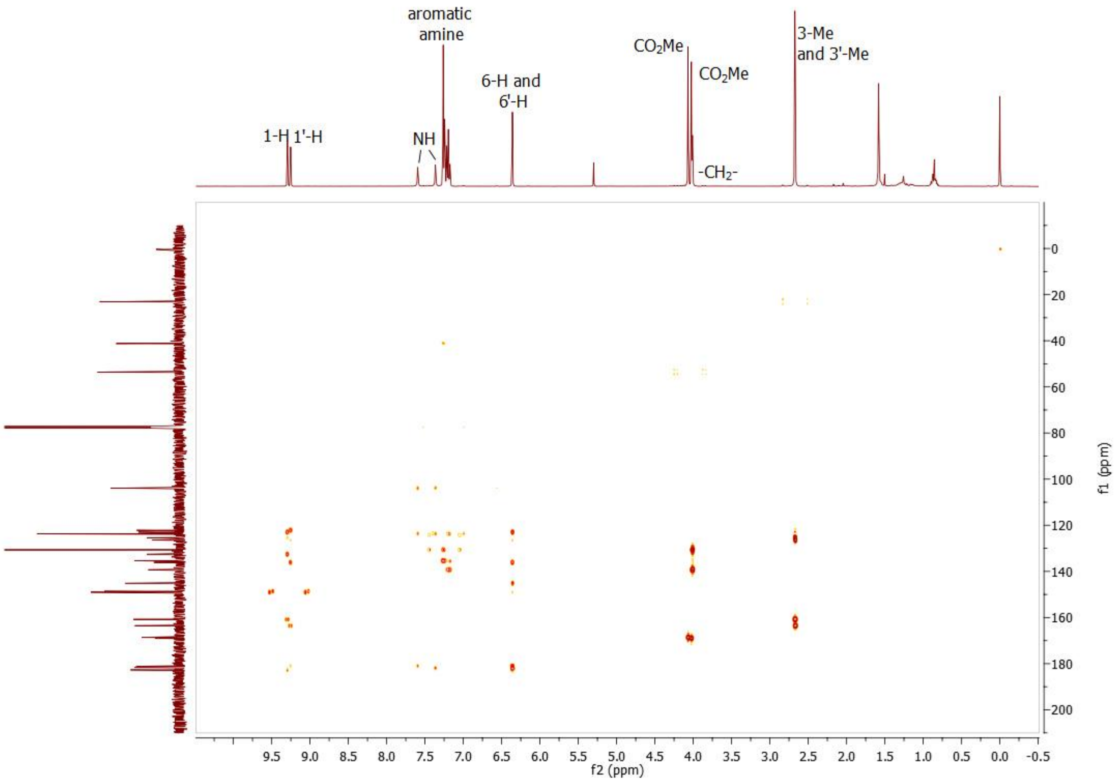

Figure S9. HMBC spectrum of compound **15**.

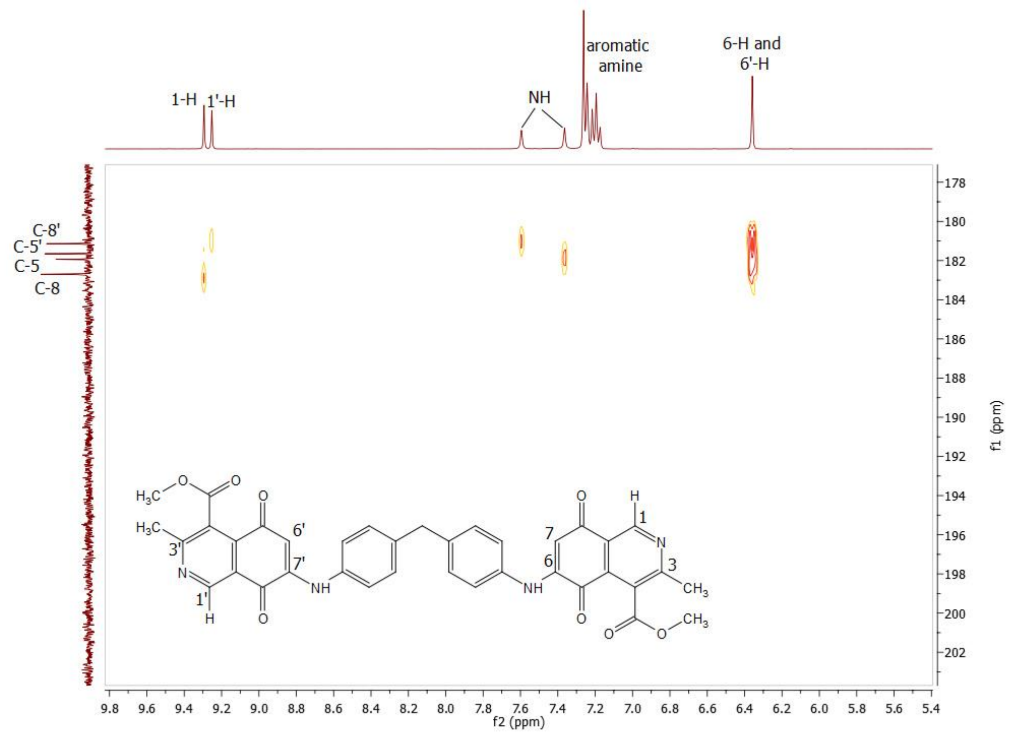

Figure S10. Expanded HMBC spectrum view of compound **15**.

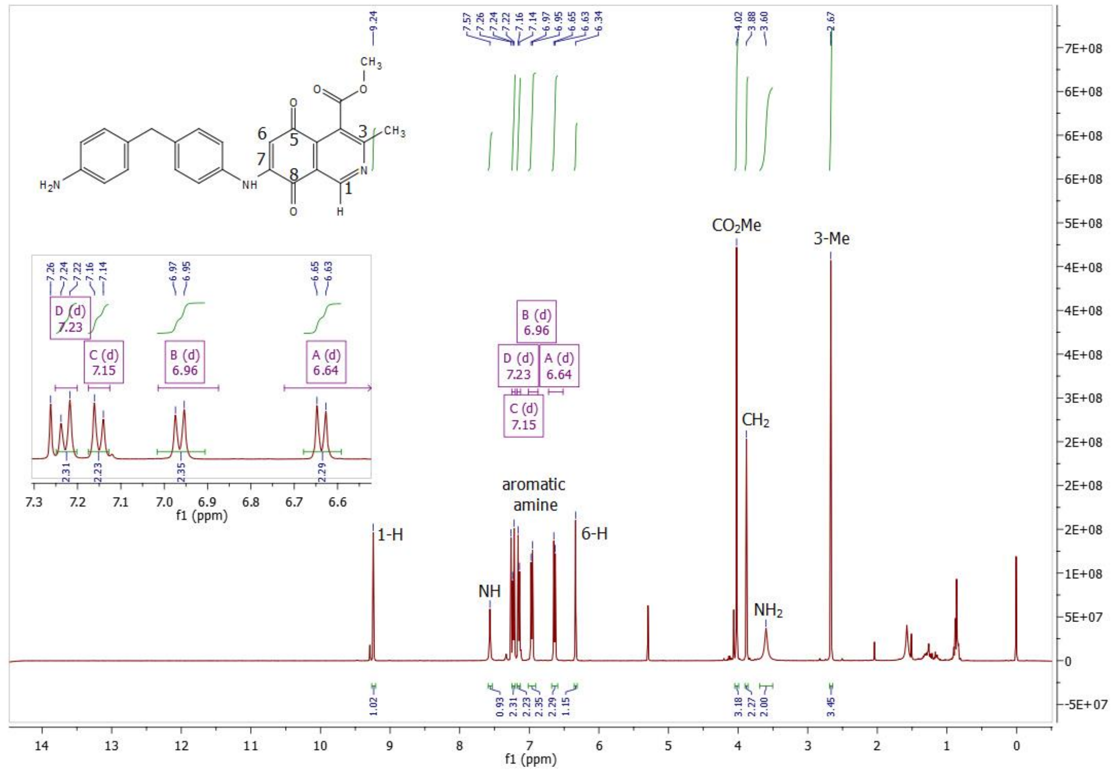

Figure S11. ^{1}H-NMR spectrum of compound **8**.

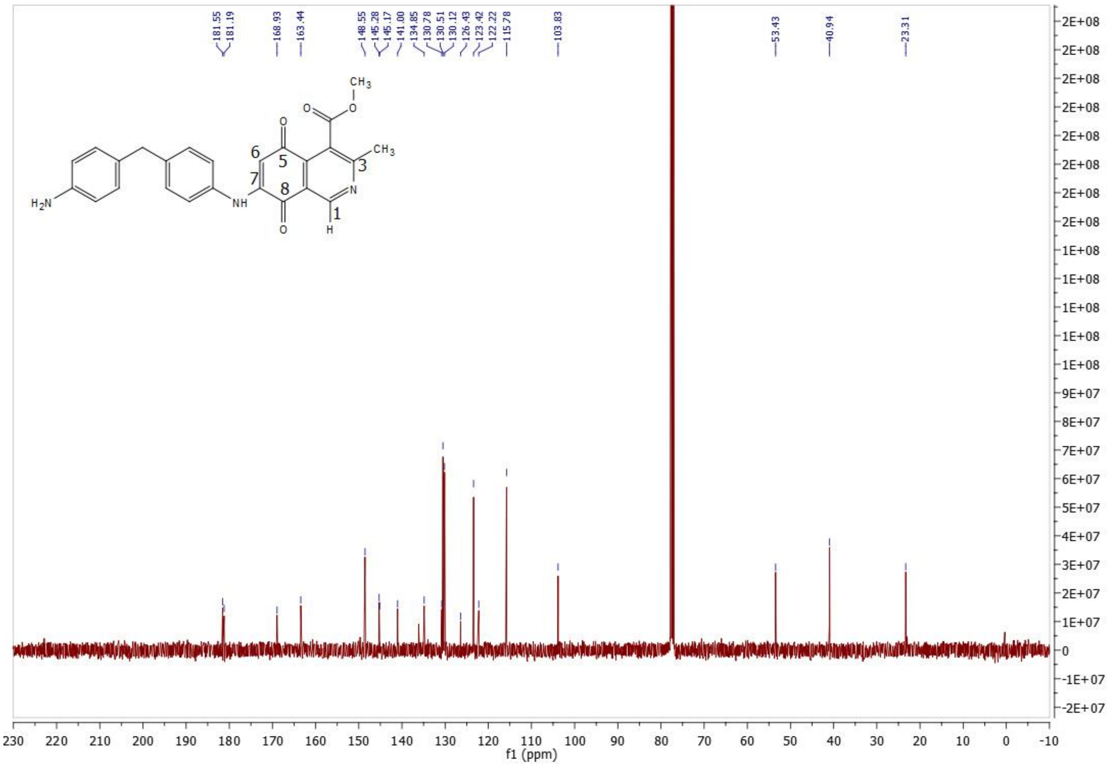

Figure S12. ^{13}C-NMR spectrum of compound **8**.

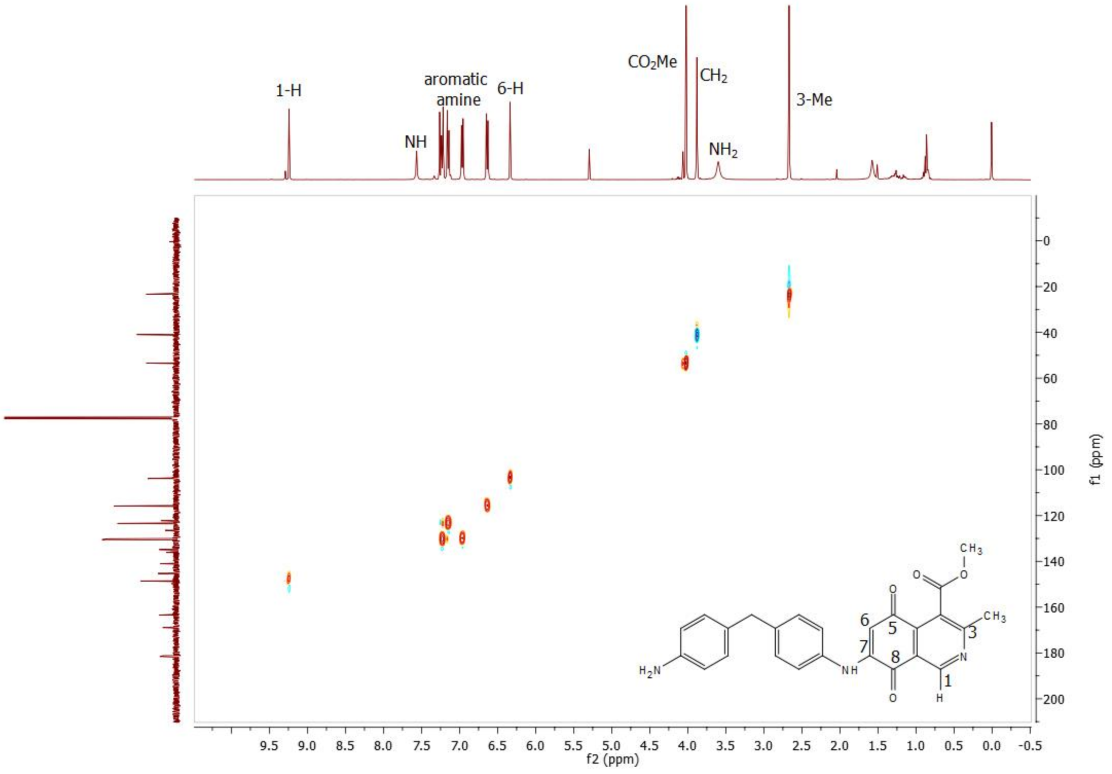

Figure S13. HSQC spectrum of compound **8**.

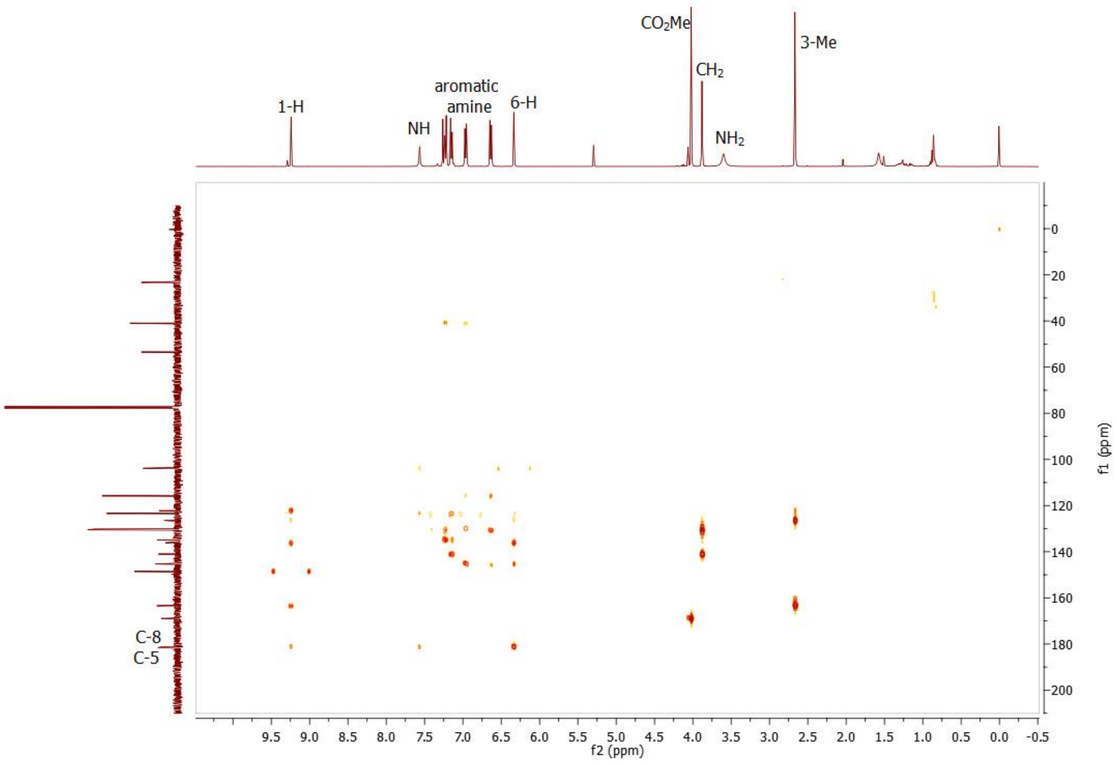

Figure S14. HMBC spectrum of compound **8**.

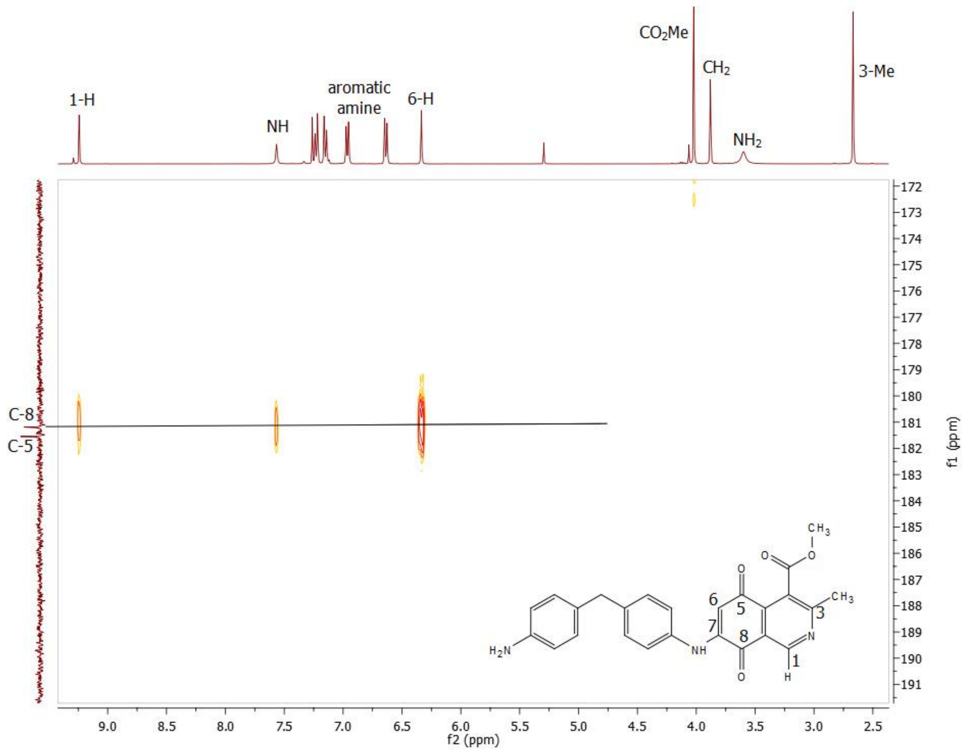

Figure S15. Expanded HMBC spectrum view of compound **8**.

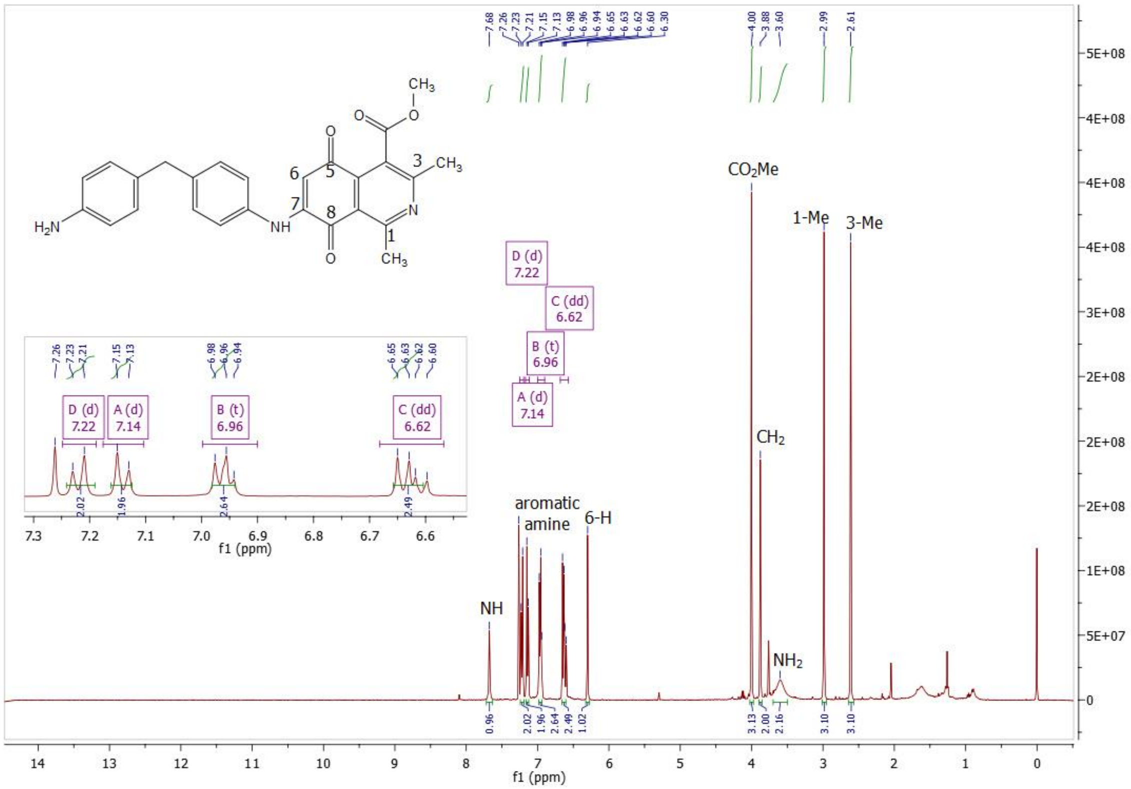

Figure S16. ^{1}H-NMR spectrum of compound **6**.

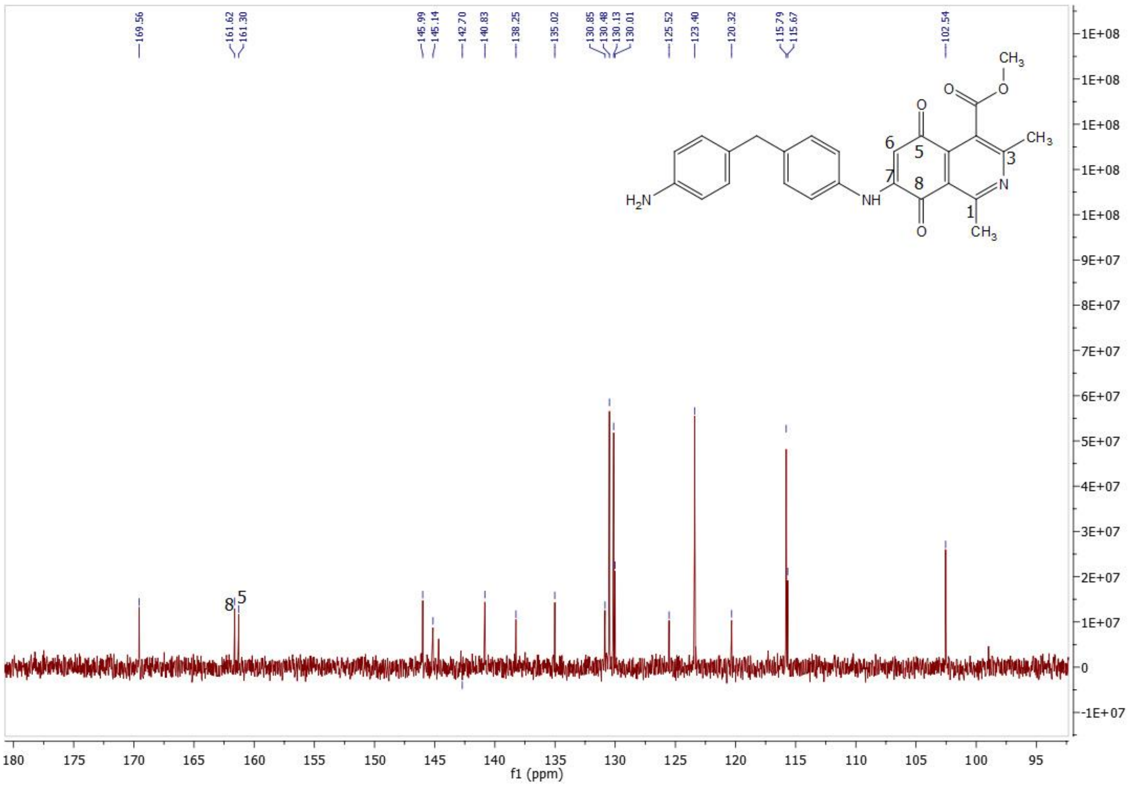

Figure S17. ^{13}C-NMR spectrum of compound **6**.

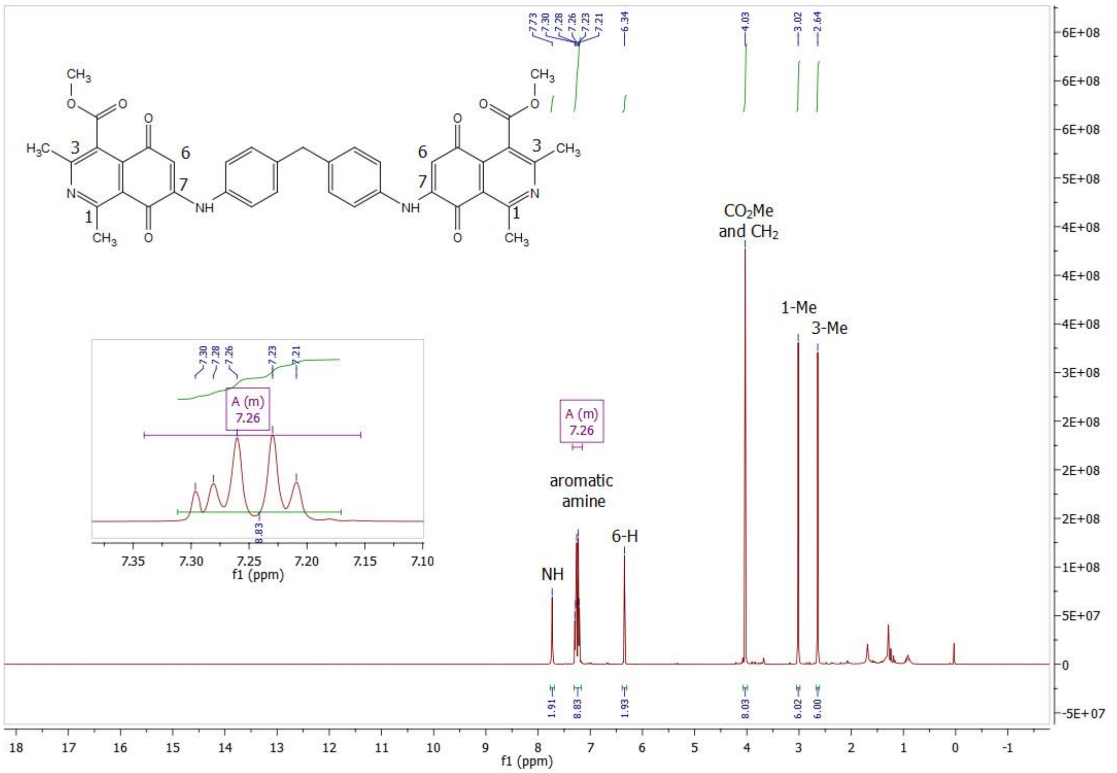

Figure S18. ^{1}H-NMR spectrum of compound **11**.

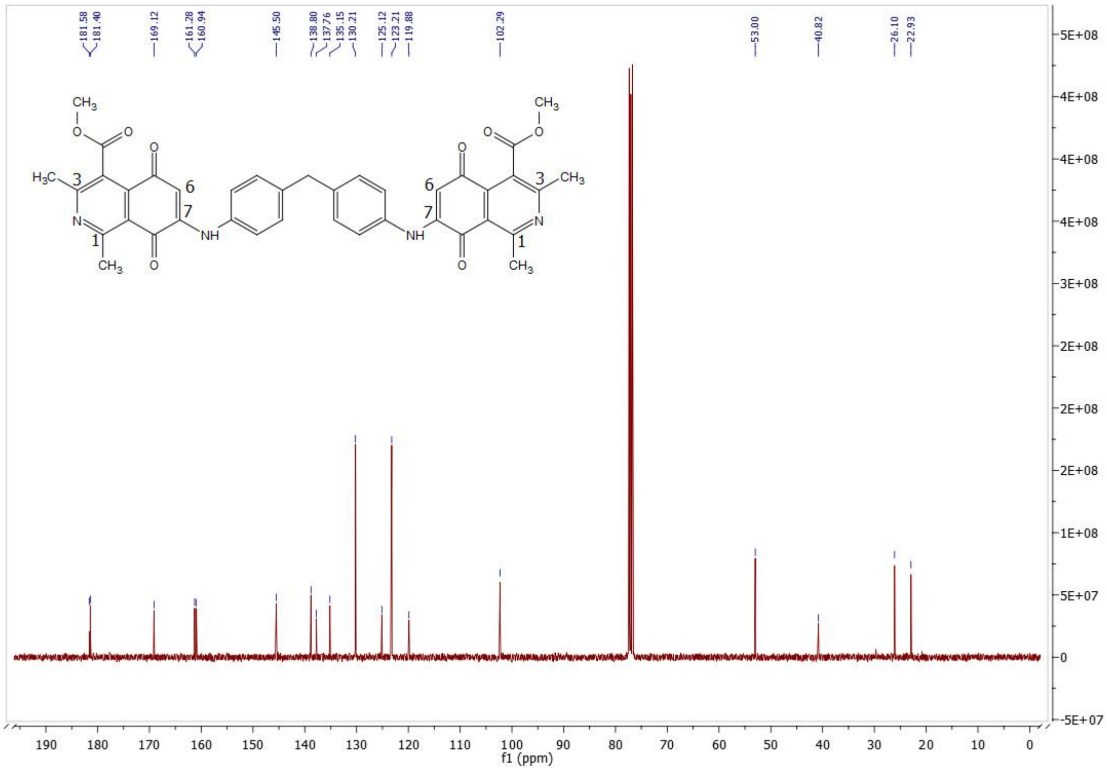

Figure S19. ^{13}C-NMR spectrum of compound **11**.

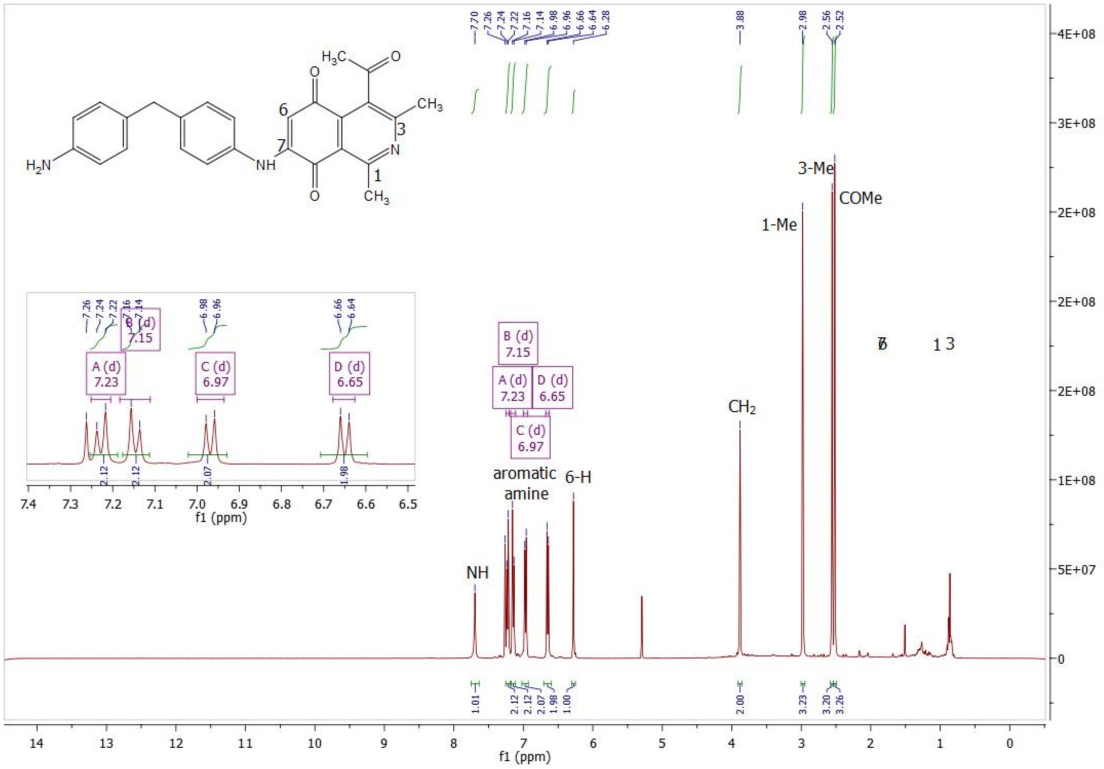

Figure S20. ^{1}H-NMR spectrum of compound **7**.

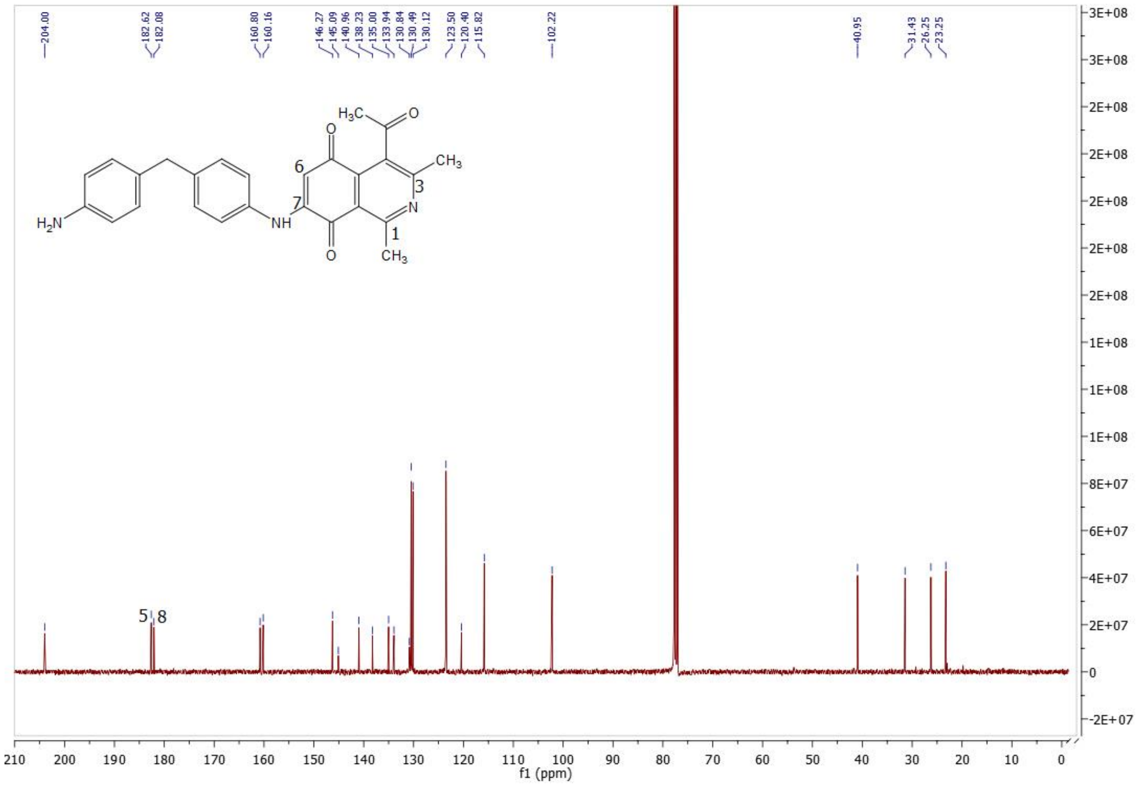

Figure S21. ^{13}C-NMR spectrum of compound **7**.

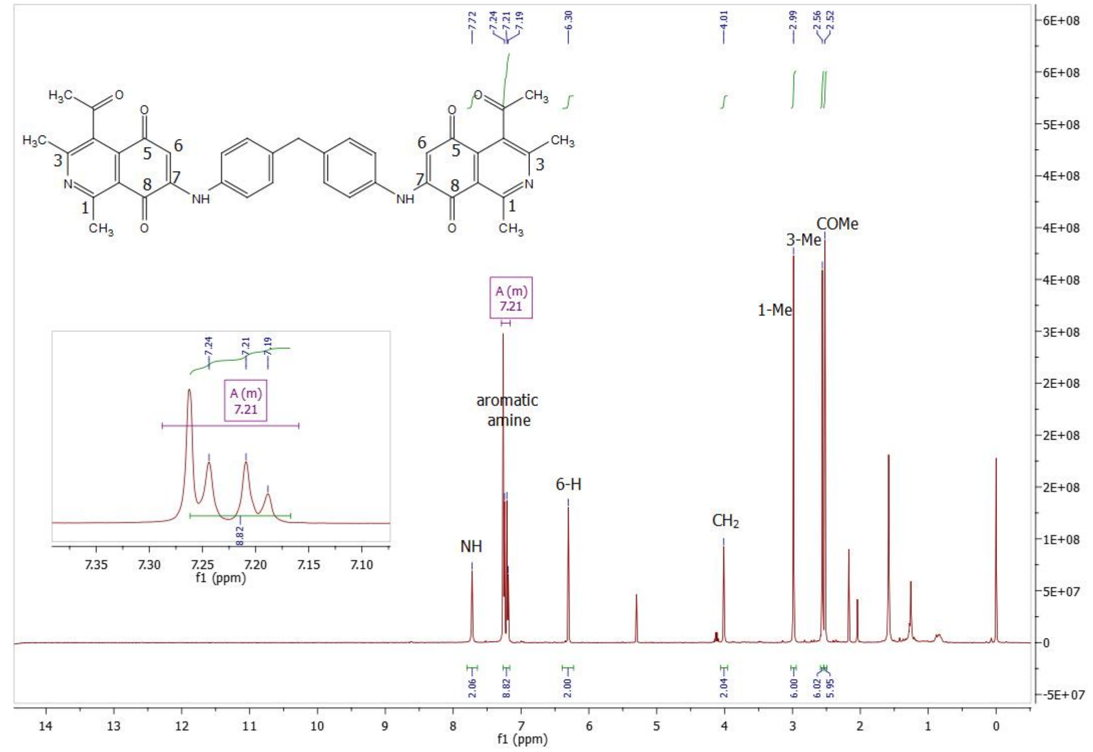

Figure S22. ^{1}H-NMR spectrum of compound **12**.

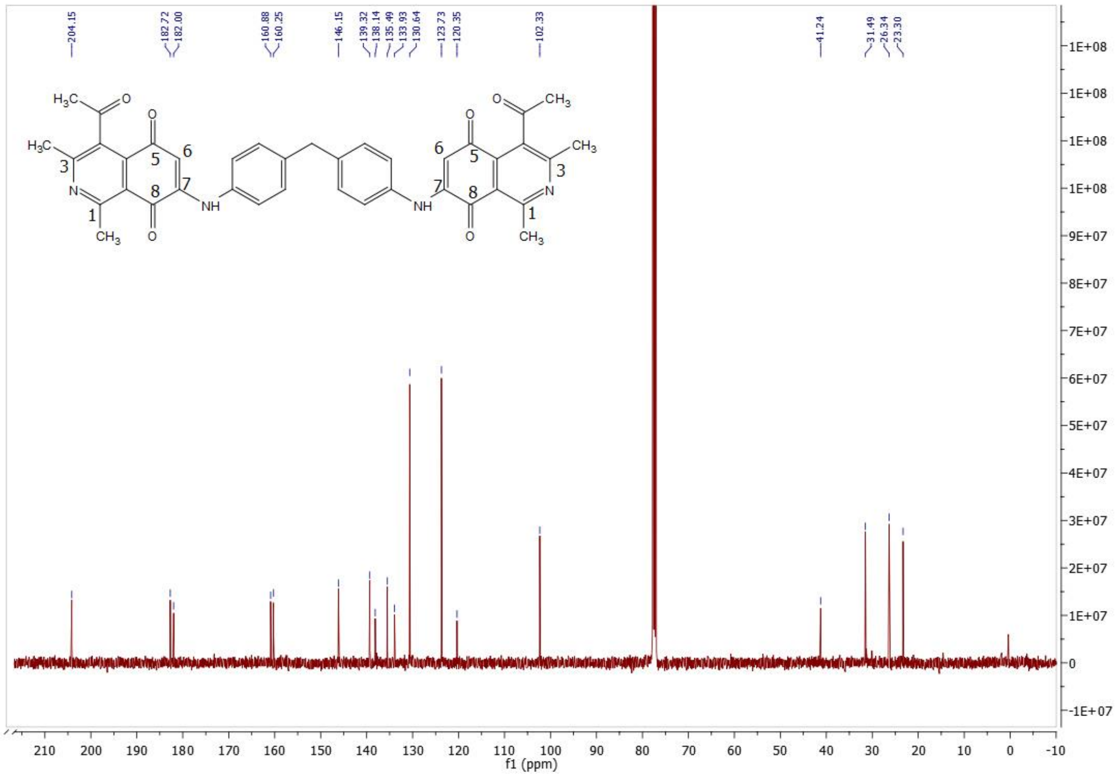

Figure S23. ^{13}C-NMR spectrum of compound **12**.

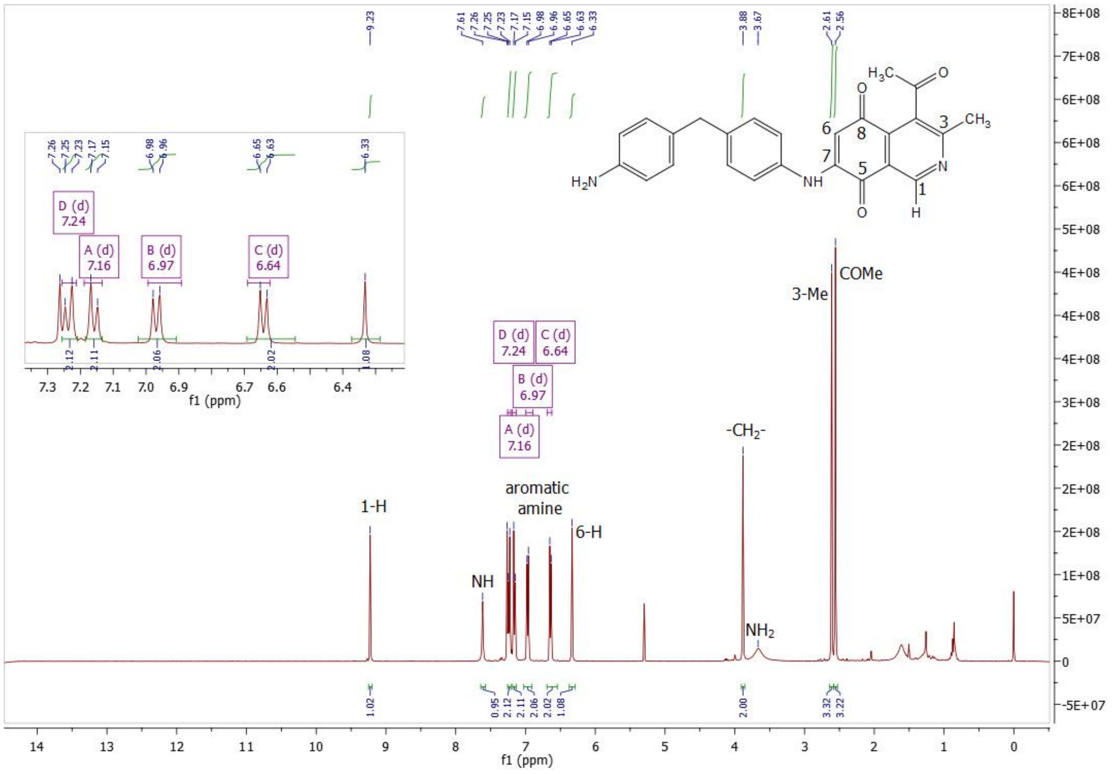

Figure S24. ^{1}H-NMR spectrum of compound **9**.

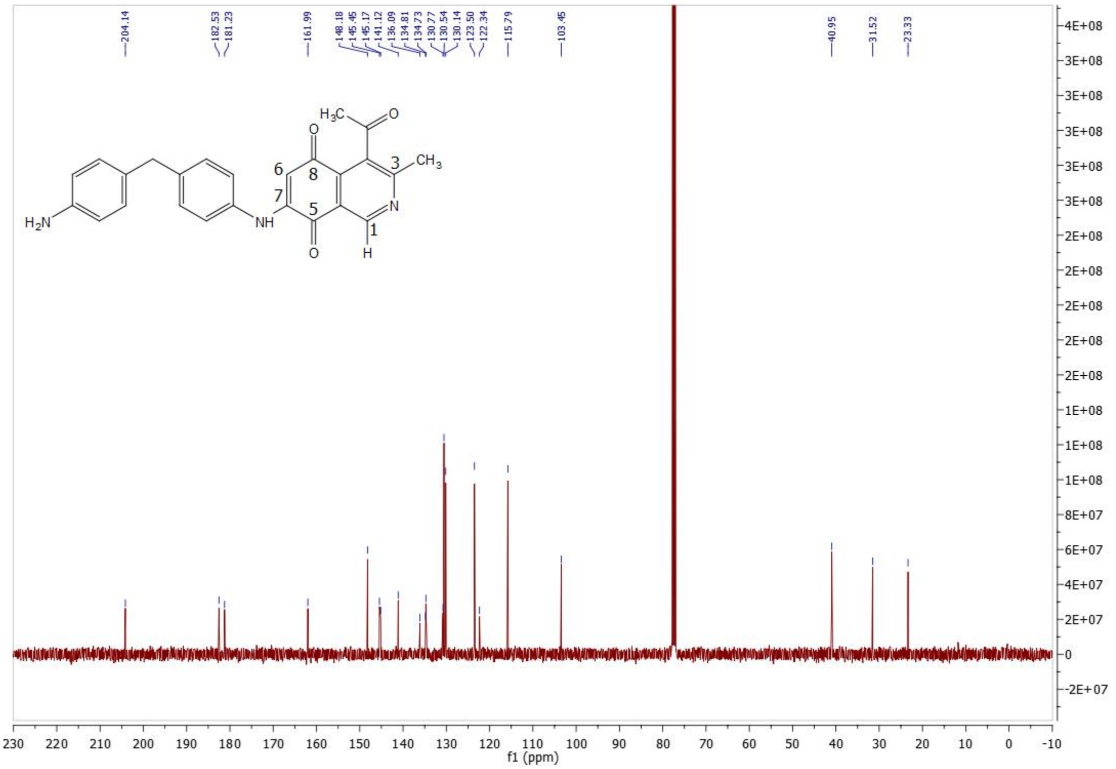

Figure S25. ^{13}C- NMR spectrum of compound **9**.

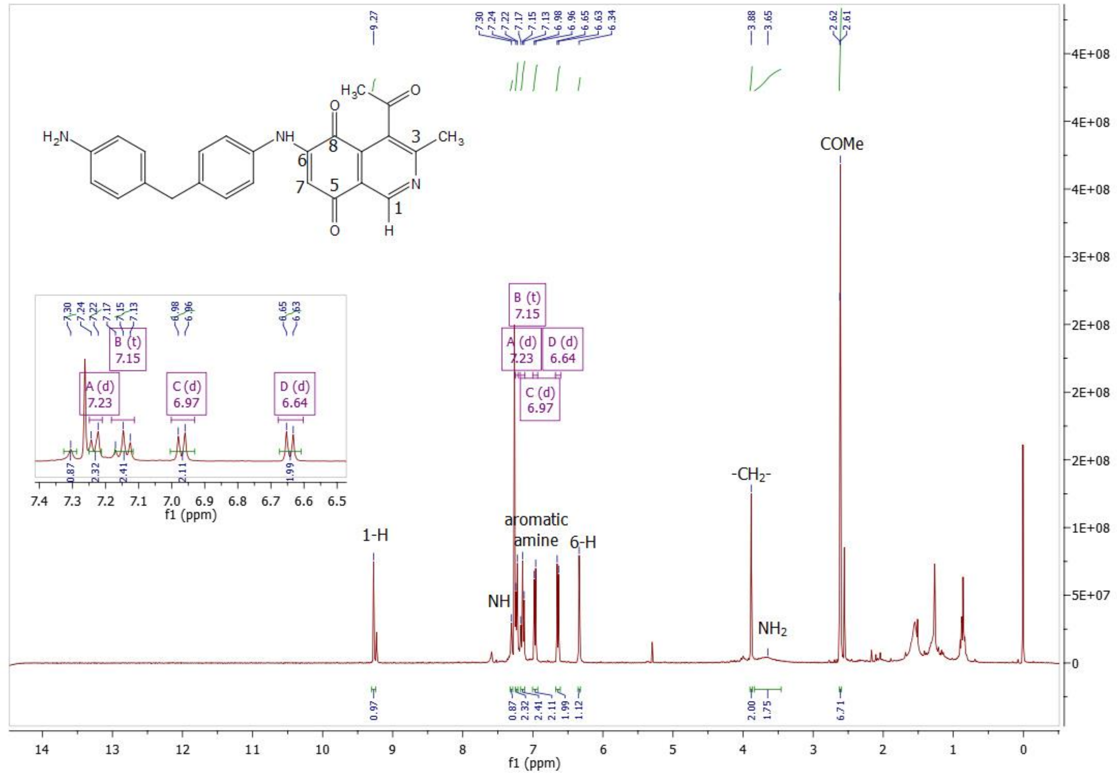

Figure S26. ^{1}H-NMR spectrum of compound **10**.

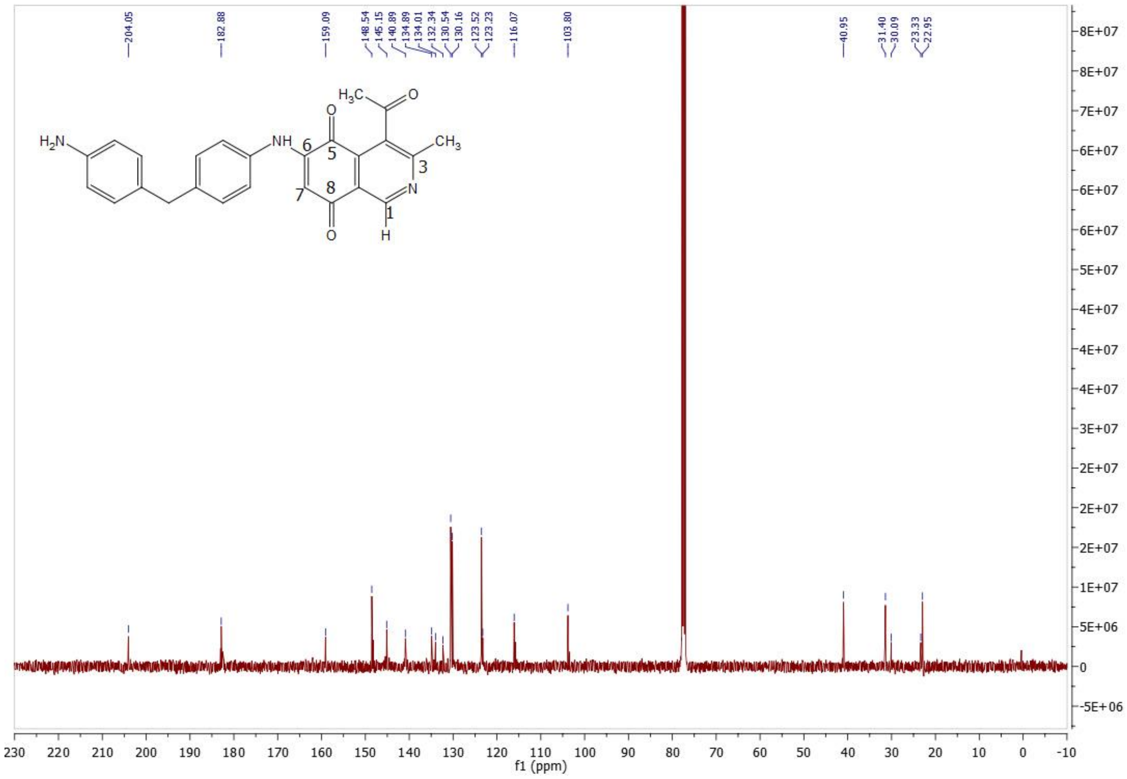

Figure S27. ^{13}C-NMR spectrum of compound **10**.

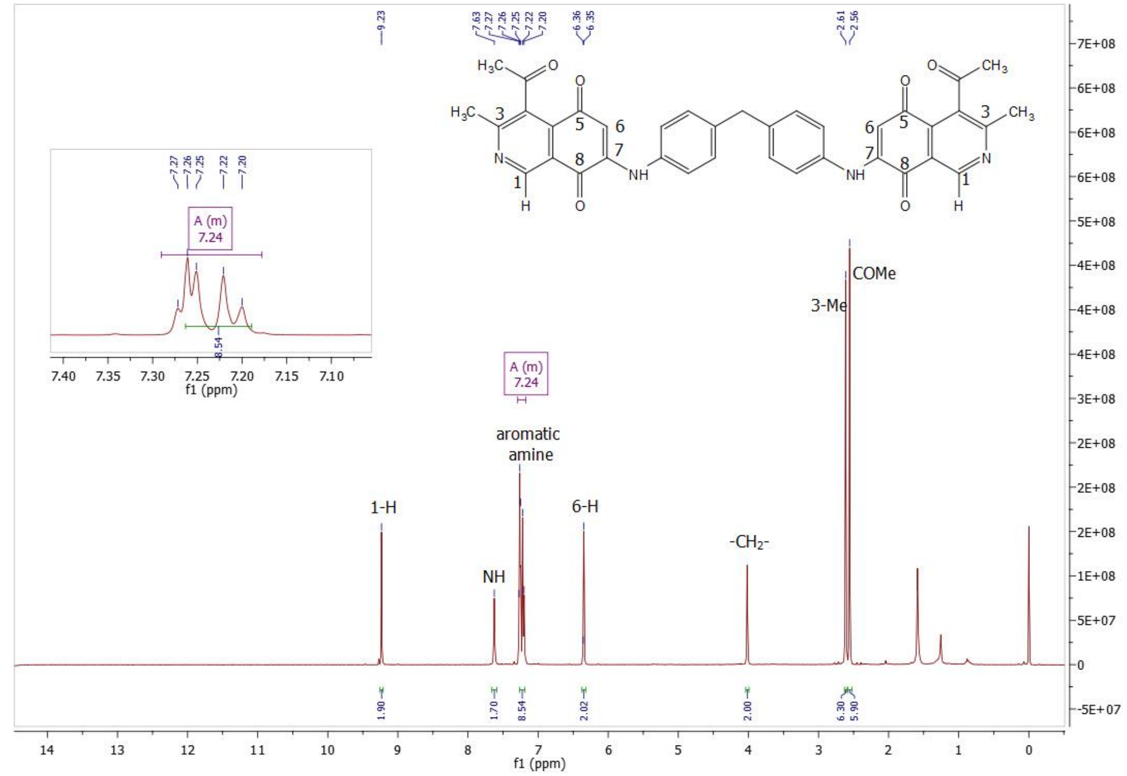

Figure S28. ^{1}H-NMR spectrum of compound **14**

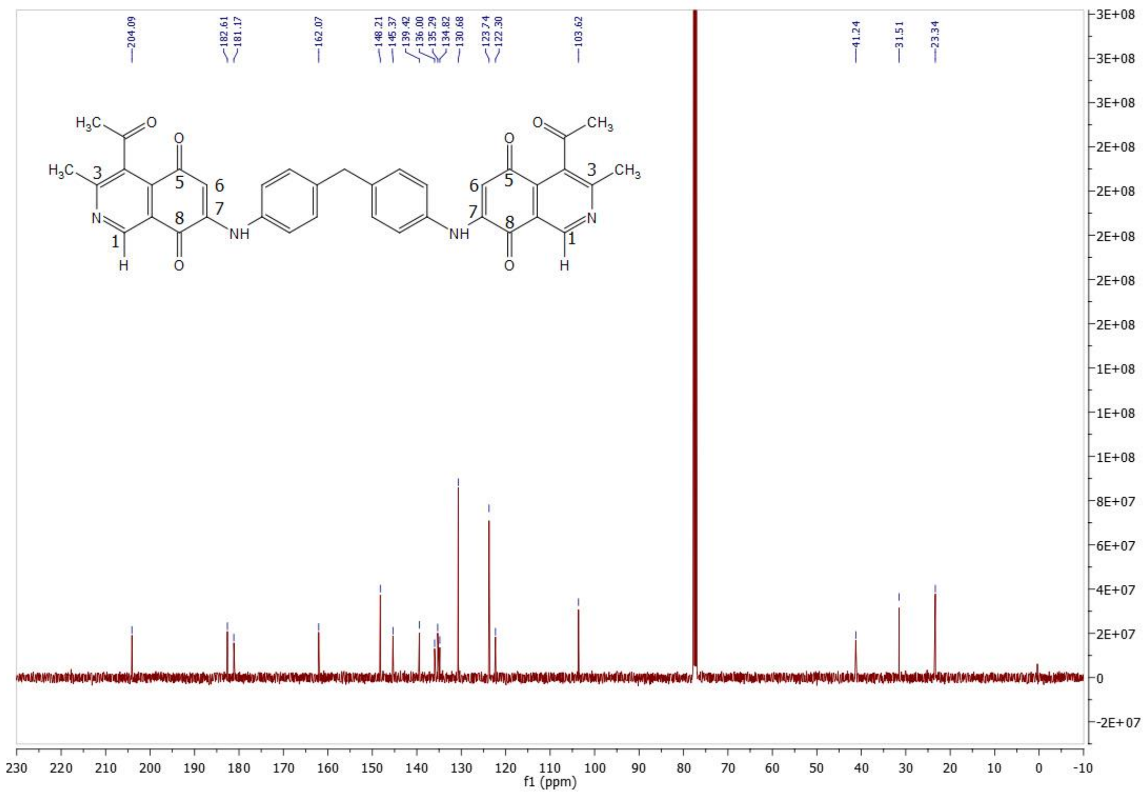

Figure S29. ^{13}C-NMR spectrum of compound **14**.

MASS MEASUREMENT ERROR

			calculated	found	mass measurement error		
	M	H+	(M+H)+	(M+H)+	(ppm)		
6	441.1689	1.0073	442.1762	442.1761	-0.181		
7	425.1739	1.0073	426.1812	426.1798	-3.238		
8	427.1532	1.0073	428.1605	428.1595	-2.289		
9	411.1583	1.0073	412.1656	412.1649	-1.650		
10	411.1583	1.0073	412.1656	412.1661	1.262		
11	684.222	1.0073	685.2293	685.2208	-12.404		
12	652.2322	1.0073	653.2395	653.2365	-4.592		
13	656.1907	1.0073	657.1980	657.1965	-2.282		
14	624.2009	1.0073	625.2082	625.2083	0.192		
15	656.1907	1.0073	657.1980	657.1991	1.704		
